# Purified Anthocyanins Indicated No Significant Effect on Arterial Stiffness, Four-Limb Blood Pressures and Cardiovascular Risk—A 12-Week Dose–Response Trial in Chinese Middle-Aged and Elderly Adults with Hyperglycemia

**DOI:** 10.3390/nu18010112

**Published:** 2025-12-29

**Authors:** Zhaomin Liu, Minmin Li, Yuming Chen, Cheng Wang, Jianyin Chen, Huanhuan Long, Ruqing Liu, Jiachi Chiou, Chaogang Chen

**Affiliations:** 1 Department of Nutrition, School of Public Health, Sun Yat-sen University (North Campus), Guangzhou 510080, China; 2 Department of Clinical Nutrition, Sun Yat-Sen Memorial Hospital, Guangzhou 510080, China; 3 Guangzhou Bai-Yun District Community Health-Care Center, Guangzhou 510080, China; 4 Faculty of Preventive Medicine, School of Public Health, Sun Yat-sen University, Guangzhou 510080, China; 5 Department of Food Science and Nutrition, The Hong Kong Polytechnic University, Hung Hom, Kowloon, Hong Kong 999077, China

**Keywords:** randomized controlled trial, anthocyanins, dose–response effect, pulse wave velocity, ankle-brachial index, cardiovascular risk

## Abstract

**Background:** Diabetes is associated with sub-optimal vascular function. Current evidence suggested purified anthocyanins (ACNs) could improve cardio-metabolic health, but the dose–response effects on arterial stiffness have not been verified. **Objectives:** To assess the dose-responsiveness of purified ACNs on vascular stiffness and cardiovascular risk among Chinese middle-aged and elderly patients with either prediabetes or early diabetes. **Methods:** This was a secondary analysis based on a 12-week double-blind, randomized and placebo-controlled trial. Eligible participants were randomly assigned to placebo, 160, 320 and 640 mg/d ACNs groups (*n* = 46/group). Information on dietary intakes and lifestyle habits and blood samples were collected at baseline and at week 12. Arterial stiffness and vascular function were measured by brachial-ankle pulse wave velocity (baPWV), ankle-brachial index (ABI) and four-limb blood pressures. Composite cardiovascular indices were estimated based on lipids and anthropometric data. **Results:** Total 184 eligible participants were recruited and 19 withdrew during the intervention. Baseline characteristics were generally comparable among groups. No significant effects or dose–response relationships were observed by ACNs supplementation on arterial stiffness and cardiovascular risk factors. **Conclusions:** The 12-week randomized controlled trial among Chinese middle-aged and elderly adults with dysglycemia showed multiple dosages of anthocyanins had no significant impacts on arterial stiffness and cardiovascular risk.

## 1. Introduction

Diabetes is a major risk factor for atherosclerosis and associated with compromised vascular function and dysregulated lipids, contributing to significant increase in cardiovascular morbidity and all-cause mortality. Compared with pharmaceutical therapies, phytochemicals are safer and more cost-effective in management of cardiometabolic conditions. Anthocyanins (ACNs) are one major subclass of flavonoids and natural plant pigments conferring purple, blue and red hues to plants. In vitro and animal experiments suggested that ACNs and their metabolites may improve vascular function through enhancing antioxidant defense, reducing oxidative stress and inflammation, augmenting nitric oxide production and bioavailability or modulating gut microbiota [1,2]. Observational studies also suggested higher intake of flavonoid or ACN-rich foods were associated with improved arterial stiffness and lowered risk of cardiovascular diseases (CVD) [3,4,5].

Despite the favorable roles suggested by laboratory and observational studies, randomized controlled trials (RCTs) by ACN intervention have reported equivocal findings on vascular health [2,6,7,8]. A recent meta-analysis of RCTs suggests that chronic berry consumption benefits endothelial function (flow-mediated dilation, FMD) but is less certain on arterial stiffness including pulse wave velocity (PWV) and augmentation index (AIx) [8]. A systematic review supported an improvement on arterial stiffness by flavonoids intake [4], but another found null improvement by chronic ACNs supplementation [6]. The inconsistencies might be due to the wide heterogeneity in ACNs formula and dosage, the food matrix, durations and participants’ features. More clinical trials are required to clarify the efficacy and determine the optimal dosage of ACNs on vascular distensibility.

PWV has been established as a gold standard tool for arterial stiffness and a promising biomarker in cardiovascular diagnostics [9]. Studies confirmed that both PWV [10] and ankle-brachial index (ABI) [11] independently predict all-cause mortality or major cardiovascular events in diabetic patients. A combination of high PWV and low ABI exhibited superior associations with health outcomes in diabetic patients [12] and all-cause mortality in the elder population [13]. Furthermore, four-limb blood pressures are recommended for early detection and diagnosis of hypertension. Increased systolic inter-arm (sIAD) and inter-ankle (sIAND) differences in blood pressure are thought to be associated with atherosclerotic vascular damage in the systemic arterial tree [14]. In addition, both sIAD and sIAND are well-correlated with arterial stiffness and considered as the valid measures for vascular function to assess preclinical vascular diseases [15] and predict CVD events [16] and all-cause mortality [17].

There was considerable inconsistency in the evidence on the efficacy of ACNs with vascular function. No study has testified the dose-responsiveness of ACN on arterial stiffness and four-limb BPs, and its optimal dosage on vascular function has not been determined especially in at-risk populations. We thus conducted secondary analysis on the basis of a 12-week RCT which was conducted among 184 Chinese middle-aged and elderly adults with either prediabetes or early diabetes to testify the dose–response effects of purified ACNs (0, 160, 320 and 640 mg/d) on vascular function and composite CVD variables. We hypothesized that patients with dysglycemia under higher dosages of ACNs supplementation would have better improved arterial elasticity and CVD health.

## 2. Materials and Methods

### 2.1. Participants Recruitment

The study was based on a 12-week randomized, double-blind, placebo-controlled, dose–response trial which was conducted from October 2021 to February 2024 with the original purpose to testify the dose–response effects of ACNs on glycemic control and insulin sensitivity. Participants were recruited from health check-up center or local communities. Potential participants were screened for their initial eligibility by questionnaire via either telephone or in person interview. Persons who were identified at high risk of type 2 diabetes (i.e., a recent medical record of hyperglycemia, metabolic syndromes, medical or family history of diabetes, etc.) were invited to receive a standard 75 g oral glucose tolerance test (OGTT) for diabetes determination. For prediabetes, impaired fasting glucose was defined as fasting glucose (FG) within 5.6 to 6.9 mmol/L and impaired glucose tolerance was defined as 2 h post-load glucose (2 h PG) within 7.8 to 11.0 mmol/L. Diabetes was defined as FG level ≥ 7.0 mmol/L or 2 h PG level ≥ 11.1 mmol/L [18].

The Biomedical Ethics Committee of School of Public Health, Sun Yat-sen University approved the study protocol (No. 042 on 30 March 2020). Written informed consents have been obtained from all participants before enrollment. The trial has been registered at Chinese Clinical Trial Registry (ChiCTR2100041743) before initiation of recruitment.

### 2.2. Inclusion and Exclusion Criteria

Participants were eligible if they were aged 45 to 75 years; with a fasting glucose (FG) ≥ 5.6 mmol/L or a 2 h post-load glucose (PG) ≥ 7.8 mmol/L by standard OGTT; a body mass index (BMI) within 18.5 to 35 kg/m^2^. They were excluded if they had severe cardiovascular, endocrinal, hepatic or renal dysfunction in the prior 6 months; any malignancies in the last 5 years; abnormal vaginal bleeding for postmenopausal women; were a heavy smoker (>1 cigarette/d) or a heavy alcohol drinker (>30 g alcohol/d); taking medications for losing weight or long-term antibiotic users; mental or cognitive disorders; allergic to berries. Patients of clinically diagnosed type 2 diabetes or hyperlipidemia were allowed if they were undertaking a stable dosage and mono-therapy managed by a physician in the prior 3 months and across the intervention. All the eligible participants were asked to attend an orientation talk during which detailed study information and requirements were introduced.

### 2.3. Supplements, Randomization and Allocation

Both ACN (Medox) and placebo (maltodextrin) capsules were provided by Medpalett AS (Biolink Group, Sandnes, Norway). Treatment identity was masked by providing all interventions in capsules identical in weight, appearance and packaging. Each Medox capsule contains 80 mg anthocyanin citrate, which are concentrated extracts from wild bilberries (*Vaccinium myrtillus*) and blackcurrant (*Ribes nigrum*) comprising a mixture of 32% cyanidin, 58% delphinidin, 2.5% petunidin, 2.5% peonidin and 3.0% malvidin. ACN capsules also contained pullulan, maltodextrin and citric acid to maintain its stability. All capsules were packed into vacuumed blister and stored in cool, dark and dry place.

Before randomization, participants were required to take a two-week run-in exercise with intake of placebo capsules in order to familiarize them with the study requirements. Participants who consumed at least 90% assigned dosages and complied with all lifestyle instructions during the run-in phase, were randomly allocated into 4 treatment groups (0, 160, 320 and 640 mg/d ACNs) by a block randomization strategy in a block size of 8. The random numbers were computer-generated and performed by personnel not involved in the investigation. To secure blinding, participants in all the groups took the same number of 8 capsules daily. For example, participants in the 320 mg/d ACN group consumed 4 capsules of Medox and 4 capsules of placebos, while in the 160 mg/d group, they took 2 Medox and 6 placebo capsules. Total 184 eligible participants were randomized. Series numbers (1–184) were labeled in the cardboard boxes which contained a monthly dosage. Supplements were assigned to participants according to their sequence of enrollment. The group allocation was blinded to all the participants and study investigators. Allocation disclosure was allowed only if serious adverse events occurred with approval from the principle investigator. Blinding efficacy was tested at the end of the trial by asking participants to guess their assignment and comparing the correct rates among groups.

### 2.4. Instructions to Participants and Compliance Assessment

Participants were free living. They were asked to reduce ACN- or polyphenol-rich foods intake (i.e., purple potatoes, blue strawberries, brassicas, mulberries, cherries, waxberries, red or black rice, red wine and strong tea, etc.) to less than 2 times per week and to refrain from phytochemical supplements and usages of antioxidants or probiotics (i.e., sesame, flax seeds, resveratrol, vitamin E and C, fish oil, berries or berry products, etc.) since the run-in phase. Alcohol drinking was restricted to no more than 30 g alcohol/week. Participants were asked to maintain their habitual lifestyles and medications unchanged during the study course. Both telephone and personal interviews were conducted monthly by research staff to investigate the supplements’ consumption, adverse events, medication usages and lifestyle changes. Compliance was evaluated by counting the leftover capsules to estimate the percentage of intake. Good compliance was defined as intake of at least 80% of expected dosages and attendance of three clinical visits for specimens’ collection.

### 2.5. Data Collection

#### 2.5.1. Questionnaire Survey and Anthropometric Measures

Socio-demographics were investigated at baseline via face-to-face or telephone interviews using a pre-tested questionnaire. Anthropometrics including body weight, height and waist circumference were measured at baseline and at week 12 by standard protocols. Dietary intakes were evaluated by a validated 79-item food frequency questionnaire (FFQ) at baseline and at the end of trial [19]. Dietary energy and nutrients intakes were calculated using the 2018 Chinese Food Composition Table [20]. Consumption of major nutrients and food groups as well as the changes across intervention were compared among different groups. Physical activities (PAs) were evaluated by a short form of the International Physical Activity Questionnaire (IPAQ) to investigate the weekly time spent on sedentary, walking, moderate and intensive activities [21]. Total PA energy expenditure was expressed as MET-h per week (metabolic equivalent of task hours per week) according to an established compendium PA coding scheme [22]. Basal and final PA as well as their pre–post changes were compared among groups.

#### 2.5.2. Arterial Stiffness and Four-Limb Blood Pressures

Brachial-ankle PWV (baPWV) and ABI were measured in both upper and lower limbs by trained research staff using a non-invasive vascular screening device of Omron Colin BP-203RPEIII (Omron Healthcare, Kyoto, Japan) based on a standard protocol [23]. The measures were made with participants in supine position after a 10 min rest. Participants were asked to refrain from coffee, alcohol and intensive exercise for at least 12 h and to empty their bladder before testing. They were asked to keep calm and remain immobile during measurement. Standard cuffs with oscillometric sensors were attached to their upper arms and ankles. One heart sound detector was placed at the left edge of the sternum. Pulse waves of bilateral brachial and posterior tibial arteries were recorded simultaneously over 10–15 consecutive heartbeats. BaPWV was estimated by dividing the transmission distance (cm) with the time interval (s) from brachium to ankle. The system automatically and simultaneously measured BPs in all four limbs. ABI was calculated as the SBP ratio in the ankle to the brachial artery. Maximum PWV (the higher lateral baPWV) and minimum ABI (the ratio of lower ankle SBP to higher brachial SBP) were further estimated. Systolic inter-arm and inter-ankle blood pressures (sIAD and sIAND) were calculated accordingly.

#### 2.5.3. Specimens Collection and Biochemical Testing

Blood collection was made at the start of run-in, week 0 and week 12 of intervention. Overnight fasting venous samples were collected in both NaF, EDTA and blank tubes. Plasma and serum were separated within 2 h after blood collection and centrifuged at 3000 r/min for 15 min at 4 °C. Samples were allocated into several vials and stored at −80 °C in a freezer until analysis. Blood samples which were collected at baseline and at the end of the trial were analyzed simultaneously in the same run of the auto-analyzer. Lipids including total cholesterol (TC), triglycerides (TG), HDL and LDL-cholesterol were tested by enzymatic methods on Beckman Coulter AU5821 (Brea, CA, USA). All the intra- and inter-CVs were less than 5%. Remnant cholesterol (RC) was calculated by subtracting HDL-c and LDL-c from TC. Non-HDL was estimated by the formula of (LDL-c + 1/5 × TG). The Framingham risk score (FRS) was estimated on the basis of several common coronary risk factors (age, sex, TC, HDL-c, smoking and SBP) to assess individual’s risk of developing coronary heart disease (CHD) [24].

### 2.6. Sample Size and Power Estimation

Sample size estimation was made for the original study outcome of HbA1c using the formula of 2[(Z_α/2_ + Z_β_) × SD/δ]^2^] for two-arm RCT, with SD of 0.35% for the change in HbA1c [25]. Based on the conventional two-tailed α of 0.05 and 90% power (β = 0.10), and accounting for a 10% drop-out, 38 subjects per group are appropriate to obtain a significant change in HbA1c (δ = 0.2%). Post hoc power estimation was made for current main outcomes according to their SD and mean differences at the 3-month changes by 320 mg/d ACNs supplementation, which were 94% for max-baPWV, 95% for min-ABI, 91% for sIAD and 93% for sIAND.

### 2.7. Statistical Analysis

All statistical analyses were performed using IBM SPSS 25.0 software. Statistical significance was defined at an adjusted *p* value of 0.017 (0.05/3 due to 3 ACNs groups being compared with the placebo). Baseline characteristics were compared to examine the initial comparability among different dosage groups. A carry-forward strategy was applied for complementing the missing data at week 12 due to withdrawals. Normality was tested by Kolmogorov–Smirnov approach. Data of heterogenous variance were logarithmically transformed or analyzed by non-parametric approach. The primary analytical approach was intention-to-treat (ITT) analysis for all the participants who were randomized (*n* = 184). Per-protocol analysis was furthermore performed as sensitivity analysis among participants of good compliance (*n* = 161). Pre-post changes and change percentages were compared among groups by one-way ANOVA for ITT analysis, or analysis of covariance (ANCOVA) for per-protocol analysis with controlling for baseline variables and possible confounders. Trend analysis across different dosage groups was conducted by either parametric (ANOVA, linear trend) or non-parametric (Jonckheere–Terpstra) approach when applicable.

Further sensitivity analyses were made to testify the results’ robustness by (1) exclusion of patients who were under medications for lowering glucose, lipids and thyroid dysfunction; (2) multivariable linear mixed model (LMM) with individual participants as random factors and adjustment for baseline data to compare the 3-month change and change% among treatment groups [26]. Subgroup analyses by gender (male vs. female), age (<65 vs. ≥65 yrs), baseline BMI (<24 vs. ≥24 kg/m^2^), hyperglycemia (prediabetes vs. diabetes), hypertension (yes vs. no) and hyperlipidemia (yes vs. no) were conducted to explore possible effect modifications of ACNs treatment on the outcomes. Interactions were testified before stratification with inclusion of a product term (subgroup variable*treatment group) into the general linear models (GLM). Results by stratification were presented only for those with *p* for interactions less than 0.15 with at least one of the main outcomes.

## 3. Results

**Figure 1:**
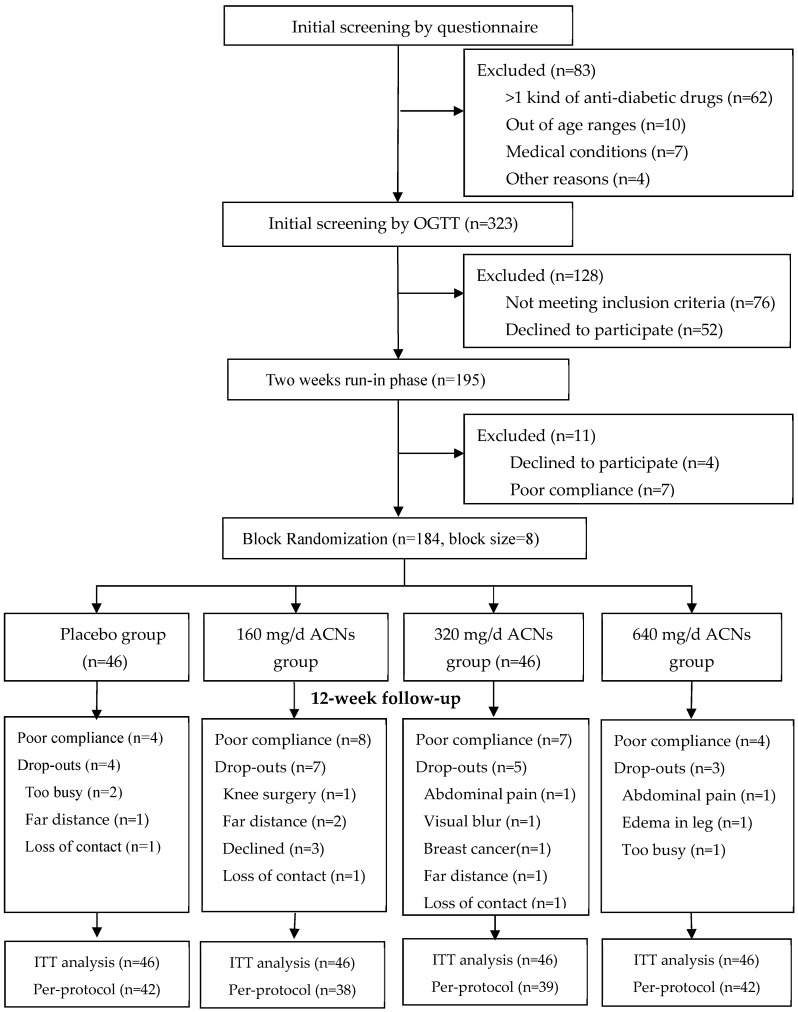
Study flow chart. **Abbreviations:** OGTT, oral glucose tolerance test; ACNs, anthocyanins; ITT, intention to treat analysis; Per-protocol, per-protocol analysis was conducted among participants who had good compliance.

The study flow was shown in Figure 1. A total of 184 participants were randomized and 19 withdrew during follow-up. The major reasons for the dropouts were discomfort or medical conditions (*n* = 6, two for abdominal pain, one for edema in foot, one for visual blur, one for diagnosis of breast cancer and one for knee operation), too busy (*n* = 3) or far distance (*n* = 3). The dropout rates were similar among different dosage groups (*p* = 0.561). In total, 161 participants (87.5%) were defined as having good compliance. The blinding tests at the final visit suggested a general good blinding efficacy (*p* = 0.865 for kappa agreement test).

Baseline features of participants were generally comparable among different dosage groups in terms of age, gender, education attainment, marital status, medication usages, weight, BMI, smoking, alcohol, and tea and coffee drinking (Table 1). Dietary intakes, sedentary time and total PA energy expenditure at baseline and at week 12 and their changes across intervention did not differ significantly among the four treatment groups (Table 2). Results by ITT analysis among 184 randomized participants demonstrated generally non-significant differences among four groups in both change and change% for variables of arterial stiffness including bilateral baPWV and ABI, the max-baPWV and the min-ABI (Table 3). Different dosages of ACNs had no significant impact on four limbs systolic and diastolic BP, mean arterial BP (MAP), sIAN and sIAND (Table 4). Neither significant effect was found on the composite CVD markers including TG/HDL-c, triglycerides-glucose index (TyG-index), TyG-BMI, visceral adiposity index (VAI) and FRS (Table 5).

Only marginal differences were suggested in the 3-month change and change% for RC (*p* = 0.048 and 0.034) and non-HDL-c (*p* = 0.037 and 0.033) yet lacking a dose–response trend. Compared with the 160 mg ACN group, RC in the 320 mg group exhibited a significant reduction in the 3-month change with a mean difference of −0.158 ± 0.618 mmol/L (*p* = 0.011 by LSD approach). Compared with the 320 mg group, a marginal increase in RC was observed in the 640 mg group in the 3-month change and change% with a mean difference of −0.131 ± 0.061 (*p* = 0.034) mmol/L and 56.6 ± 23.3% (*p* = 0.016), respectively. For non-HDL-c, compared with placebo, an increase was observed in the 160 mg ACN group with a mean difference of 0.292 ± 0.107 mmol/L in the 3 m change (*p* = 0.007) or 7.711% ± 2.910% for change% (*p* = 0.009). In addition, patients in the 640 mg group had a marginal decrease in non-HDL-c in comparison with those of the 160 mg group, with a mean difference of −0.249 ± 0.107 mmol/L in the 3 m change (*p* = 0.021) or −7.242% ± 2.910% in change% (*p* = 0.014).

Similar findings were observed by per-protocol (Table S1 and Figures S1–S13) and LMM (Table S2) analyses; neither dosages of ACNs significantly affected vascular and CVD risk variables, although a relatively low level of RC was observed in the 320 mg group at 3 m change% while an elevated level was observed in the 640 mg group with marginal significance (*P*_overall_ = 0.018~0.051). Exclusion of patients who were taking medications for hyperglycemia, hyperlipidemia or thyroid dysfunction suggested similar findings with those of whole participants (Supplemental Table S3). Subgroup analyses stratified by different groups of gender, age, basal BMI, hyperglycemia, hypertension and hyperlipidemia exhibited generally non-significant findings and a lack of dose-effectiveness (Supplemental Tables S4–S9). Marginal differences among the four groups were observed in RC at the 3-month change% in the female (*P*_overall_ = 0.026) and per-diabetes (*P*_overall_ = 0.040) subgroups, with a reduced RC in the 320 mg group.

## 4. Discussion

### 4.1. Summary of Current Findings

The 12-week RCT among Chinese middle-aged and elderly men and women with hyperglycemia examined the dose–response effects of ACNs (0, 160, 320 and 640 mg/d) and failed to find their positive effects on the improvement of arterial stiffness, four-limb BP and CVD risk. No dose–response relationship was identified either. ACNs were generally safe even in a high dosage of 640 mg/d; however, the study did not support their anti-arthrosclerosis benefits. Although marginal improvement was suggested by 320 mg/d ACNs supplementation on RC, the one or two significant exploratory findings among the dozens of comparisons should be treated with caution and require confirmation with further research. To our knowledge, this trial was the first testifying the dose–response effects of purified ACNs on arterial stiffness and four-limb BPs. Further studies exploring the mechanisms on treatment response variability by intestinal microbiome and targeted metabolome are warranted to untangle the controversial evidence.

### 4.2. Results Explanation

Reviews on ACNs with vascular function and elasticity reported inconsistent findings [2,4,6,8,27]. Our findings align with a range of trials demonstrating non-significant changes in PWV or BPs by ACNs supplementation [28,29,30,31,32,33,34,35,36] but not all [37,38,39,40]. The contradictory findings are hard to interpret by different ACNs formulas, duration, dosages or health status of participants. This is because there were studies with longer duration than ours [32], including different ACNs dosages [32,36], ACNs contained in polyphenol-rich foods [28,29,30,31,32,33,34,35,36] or among at-risk populations [28,29,30,31,32,33,34,35,36], that still reported non-significant findings in vascular stiffness. In line with our findings, a 6-month RCT [32] examined the effect of blueberry intake (1, 0.5 and 0 cup/d, containing 364, 182 and 0 mg anthocyanin and 879, 439 and 0 mg phenolics, respectively) among 115 patients of metabolic syndromes showed that, although one cup of blueberries daily improved FMD and AIx, insulin resistance, PWV, BP, NO and overall plasma thiol status were unaffected. A 12-week RCT [35] comparing aronia whole fruit (12 mg polyphenol, 10 g berries) and extract powder (116 mg polyphenol, 75 g berries) found a significant increase in FMD over the control (maltodextrin), but no significant difference in peripheral and central BP, arterial stiffness and lipids. A 3-month RCT [28] with 60 mL tart montmorency cherries (MC) concentrate (equivalent to 68–73.5 mg ACNs and 160.8–178.8 mg total phenolics) on adults at high risk of type 2 diabetes reported null effects of MC on vascular function (PWV, FMD and BP) and metabolic variables. Another randomized, open label, cross-over trial [30] on adults with central obesity also showed, consumption of ACN-rich (50 mg ACNs/500 mL) blood-orange juice for four weeks did not affect lipoproteins, aortic BPs, carotid-femoral and brachial-ankle PWV.

In contrast, several trials reported favorable improvement on arterial stiffness by ACN-rich foods or extracts [37,38,39,40]. A 12-week RCT [37] on overweight participants reported that daily dietary intakes of 293.6 mg anthocyanins and 1370 mg total polyphenols were effective in protecting against arterial stiffness, although they had no benefit on weight loss. Another study [39] among prehypertensive participants found that 12-week Aronia berry consumption improved PWV and Aix, although there were no changes in BP, endothelial function and blood lipids. The favorable changes were possibly via modulation of gut microbiome composition and richness. Evidence suggested the intricate interplay of ACNs with other constituents in the food matrix (i.e., glucose, protein, fibers, organic acids and other polyphenolic compounds) might favor improved bioavailability, leaning benefits towards whole foods over purified foods or extracts [41,42]. The food matrix may exert a protective effect against the degradation of anthocyanins by enhancing its stability, antioxidant property and bioactivity relative to its isolated counterpart [41]. In addition, different flavonoids may generate common groups of metabolites exerting synergistic or additive impacts on vascular health [41]. Further investigations on supplement processing techniques, gut microbiota interactions, inter-individual differences in genetics, ACNs in combination with lifestyle factors or medications for enhanced efficacy might help explain the results [41].

We observed a marginal difference among groups in remnant lipoproteins at the 3-month change or change% with a relative lower level of RC in the 320 mg/d ACNs group while elevated levels were found in the 640 mg/d group. Recent evidence suggested RC [43,44] and non-HDL-c [24] might actually have stronger associations with major adverse CVD events or mortality than LDL-c did. Remnant lipoproteins are deemed as the strongest candidates among triglycerides for targeting CHD risk reduction [24]. A recent systematic review and meta-analysis [45] including 28 cohort studies showed that each 1 SD increase in RC was associated with a 67% (HR 95% CI: 1.39, 2.01) increase in risk of major adverse cardiovascular events (MACE) in a notable dose–response manner. Mendelian randomization results reinforced the existence of causal relationship, suggesting the atherosclerotic risk may be majorly driven by the direct effect of RC [46]. This is because RC has a smaller particle size than LDL-c, favoring its penetration to the endothelial barrier and contributing to the progression of atherosclerosis [47]. Non-HDL-c has been confirmed to highly correlate with atherogenic remnant lipoproteins to enhance CV risk prediction when serum triglycerides are high [24]. Our study was the first RCT examining the effects of ACNs on RC; however, contrary to our expectation, we observed a paradoxical effect on RC, non-HDL-c and triglycerides-related markers (null effect) by ACNs supplementation even if these lipid variables were highly correlated. The reasons are unclear and the paradoxical findings warrant further investigation. Future studies are required with inclusion of a larger sample size and direct measurement of RC and subclasses of HDL-c to confirm the role of ACNs on lipids metabolism.

We did not observe the obvious dose–response effects of ACNs on cardiometabolic markers as other ACNs trials did [48,49,50]. Similar to our findings, studies with higher doses of ACNs-rich foods did not exhibit greater impacts on vascular function than those of lower doses [32,36]. Inadequate sample size, insufficient dosages of ACNs and short duration of intervention might contribute to the null effect. However, compared with trials that reported the significant effects of ACNs on vascular stiffness [37,38,40], our study had relatively large sample size and long enough duration. Post hoc power estimation suggested adequate power (all > 90%) for PWV-, ABI- and peripheral BP-related variables. In addition, our trial included a wide range of ACNs dosages from 0 to 640 mg/d. The insufficient dosage and duration as well as the small sample size thus seem unlikely to be driving the non-significant findings on vascular stiffness.

The health status of participants or their use of medications might confound the results. Although the current study was specifically conducted among patients with either prediabetes or early diabetes, they had generally normal vascular stiffness and BP levels and much lower rates of lipid-lowering therapy among participants. Concurrent medication therapies for metabolic conditions might affect the intervention efficacy. However, all the participants who were undertaking medications for metabolic disorders kept stable dosages and monotherapy across the intervention. Sensitivity analysis with exclusion of patients under medications and subgroup analyses by different metabolic conditions revealed similar findings to those of whole participants. We did not observe significant impacts on lipids other than RC. It is therefore unlikely participants’ health status or medications could explain the non-significant findings.

The null findings could also be explained as the varied responsiveness to ACNs treatment among participants. However, we did not investigate the factors affecting the interindividual variability in response to the intervention. Studies showed participants’ heterogeneities in their biological responses, including gut microbiome composition and (epi)-genetic profiles as well as the differences in the absorption, distribution, metabolism and excretion of bioactive compounds, might play an important role in the contradictory findings [51]. Identifying the main determinants of inter-individual differences and responsiveness profiles, remains essential to target specific populations and optimize the beneficial health effects of bioactive plant food [51].

### 4.3. Study Strengths and Limitations

The double-blinded, block randomized and placebo-controlled trial designed with multi-dose ACNs were the major strengths of this study. This study was the first to evaluate the dose–response effect of purified ACNs on arterial stiffness among Chinese adults with hyperglycemia. The study had strength in adequate sample size, generally good compliance, low drop-out, effective blinding and use of non-invasive measures for arterial stiffness. In addition, we allowed medication treatment for chronic metabolic conditions which was a common circumstance in the elderly population and this might facilitate the generalizability of the study and its findings. The data regarding the medication history and changes in prescriptions were fully investigated and followed across the intervention, which effectively limited the impact from medications on the outcomes. In addition, all the participants were instructed to restrict their ACNs/polyphenol-rich foods intake to no more than two times per weeks, terminate any phytochemicals or antioxidants supplements since the run-in phase and keep lifestyles unchanged throughout the intervention. Both per-protocol, subgroup and sensitivity analyses suggested robust findings. Although the study was conducted during the COVID pandemic period, which resulted in a relatively slow recruitment progress, no participants withdrew or were hospitalized due to virus infection or quarantine. Lifestyle changes and psychological stress might be brought about during the virus spread [52,53]. However, dietary intakes and physical activities investigated before and after intervention suggested their changes were comparable among different dosage groups.

The study’s limitations should be acknowledged. First, although we applied block randomization for participant assignment, the influence of different genders and BMI levels was not pre-specified. However, post hoc stratification suggested similar findings among subgroups. Second, due to resource limitation, ACNs’ bioavailability and participants’ compliance were not assessed by objective biomarkers. As most of previous ACNs trials did, compliance was not objectively evaluated by ACNs metabolites due to their low bioavailability, short half shelf-life and limited specificity in circulation. Third, arterial stiffness was assessed by baPWV not carotid-femoral PWV (cfPWV). Ba-PWV was an alternative technique for aortic PWV which was measured directly by oscillometry over the arm and ankle [54]. Longitudinal studies have revealed baPWV showed better predictivity for CVD events in Asian diabetic patients [10,55], as baPWV assesses the mechanical property covering both the large-sized central elastic and medium-sized peripheral muscular arteries [56]. In addition, compared with cfPWV, baPWV requires a simpler and faster recording procedure that is less traumatic for the patients and more practical and cost-effective [57].

## 5. Conclusions

This first dose–response trial among Chinese middle-aged and elder adults with dysglycemia suggested ACNs supplementation for 12 weeks had no significant effects on vascular stiffness, four-limb BP and CVD risk. Although the findings were generally non-significant, ACNs were shown to be safe, well-tolerated and had no deleterious effects on vascular health. Further investigations exploring the mechanisms of individual variability in response to ACNs by gut metabolites and targeted metabolomics are necessary.

## Figures and Tables

**Table 1:** Baseline characteristics of participants by different dosages of anthocyanins (*n* = 184).

	Anthocyanins Supplementation by Different Dosages (*n* = 46/Group)				
	Placebo	160 mg/d	320 mg/d	640 mg/d	*p*
Age, years	62.9 ± 8.0	63.8 ± 8.7	62.8 ± 8.9	60.2 ± 9.4	0.218
Female, %	37 (29.8%)	27 (21.6%)	28 (22.4%)	33 (26.4%)	0.091
Diabetes, %	26 (27.4%)	23 (24.2%)	23 (24.2%)	23 (24.2%)	0.899
Prediabetes (sIFG/sIGT/both), %	7/5/8	5/9/9	4/7/12	5/8/10	0.832
Education, college and above, %	9 (19.6%)	13 (28.3%)	12 (26.1%)	15 (32.6%)	0.607
Marriage, singled, %	11 (23.9%)	5 (10.9%)	10 (21.7%)	5 (10.9%)	0.110
Baseline hyperlipidemia, %	26 (28.6%)	16 (17.6%)	26 (28.6%)	23 (25.3%)	0.121
Baseline hypertension, %	19 (26.4%)	18 (25.0%)	20 (27.8%)	15 (20.8%)	0.734
Baseline overweight, %	30 (30.9%)	24 (24.8%)	23 (23.7%)	20 (20.6%)	0.203
Medications at baseline, %					
Diabetes	3 (6.5%)	8 (17.4%)	4 (8.7%)	4 (8.7%)	0.326
Hypertension	13 (28.3%)	13 (28.3%)	16 (34.8%)	8 (17.4%)	0.074
Hyperlipidemia	1 (2.2%)	1 (2.2%)	0	1 (2.2%)	1.000
Thyroid dysfunction	5 (10.9%)	0	3 (6.5%)	5 (10.9%)	0.146
Smoking, %	3 (6.5%)	3 (6.5%)	2 (4.3%)	2 (4.3%)	0.935
Alcohol drinking, %	20 (43.5%)	11 (23.9%)	14 (30.4%)	21 (45.7%)	0.089
Tea drinking, %	39 (84.8%)	39 (84.8%)	38 (82.6%)	40 (87.0%)	0.953
Coffee drinking, %	16 (34.8%)	7 (15.2%)	15 (32.6%)	11 (23.9%)	0.130
Anthropometrics					
Weight, kg	64.0 ± 9.5	61.5 ± 10.0	64.8 ± 11.6	60.5 ± 12.8	0.205
BMI, kg/m^2^	25.1 ± 2.9	23.9 ± 2.9	24.5 ± 3.3	23.6 ± 3.6	0.120
WHR	0.890 ± 0.058	0.897 ± 0.058	0.900 ± 0.057	0.880 ± 0.062	0.417
SBP, mmHg	129.5 ± 16.5	128.2 ± 17.4	131.3 ± 17.8	130.0 ± 18.8	0.865
DBP, mmHg	80.7 ± 11.5	77.9 ± 10.8	81.1 ± 10.4	82.2 ± 10.2	0.277
Max baPWV > 1800 cm/s, %	13 (30.2%)	11 (25.6%)	12 (27.9%)	7 (16.3%)	0.501
Min ABI < 0.9	0	0	0	0	NA
sIAD > 15 mmHg, %	0	2 (33.3%)	2 (33.3%)	2 (33.3%)	0.613
sIAND > 15 mmHg, %	0	4 (44.4%)	3 (33.3%)	2 (22.2%)	0.233

Data were presented as mean ± SD for continuous variables and compared by ANOVA, or *n* (%) for categorical variables and compared by chi-square test. Abbreviations: sIFG, sole impaired fasting glucose; sIGT, sole impaired glucose tolerance; both, having both IFG and IGT; WHR: waist-to-hip ratio; BMI, body mass index; SBP/DBP: systolic/diastolic blood pressure; baPWV, brachial-ankle pulse wave velocity; ABI, ankle brachial index; sIAD, systolic inter-arm difference; sIAND, systolic inter-ankle difference. The maximum baPWV was determined by the higher either left or right PWV. The minimal ABI was estimated by dividing the lower one of ankle SBP with the higher one of brachial SBP.

**Table 2:** Dietary intakes of major nutrients and food groups, sedentary time and total physical activities across intervention by different treatment groups, intention to treat analysis (*n* = 184).

	Anthocyanins Supplementation by Different Dosages (*n* = 46/Group)				
	0 mg/d (Placebo)	160 mg/d	320 mg/d	640 mg/d	*p*
Nutrients					
Total energy, kcal/d					
baseline	1884 ± 709	1913 ± 681	2042 ± 741	1963 ± 927	0.179
week 12	1774 ± 787	1927 ± 702	1907 ± 699	1839 ± 731	0.257
change	−109 ± 564	10 ± 782	−136 ± 654	−131 ± 748	0.920
Protein, % total energy					
baseline	21.2 ± 4.1	21.6 ± 5.4	21.1 ± 4.4	22.4 ± 4.5	0.531
week 12	20.4 ± 4.3	21.7 ± 3.7	20.8 ± 4.4	22.7 ± 4.1	0.036
change	−0.8 ± 4.2	0.1 ± 5.5	−0.3 ± 3.9	0.4 ± 5.3	0.666
Total fat, % total energy					
baseline *	23.2 (20.1, 29.4)	22.6 (17.3, 28.2)	22.0 (18.2, 26.7)	22.5 (18.4, 24.4)	0.707
week 12	23.5 ± 8.8	22.9 ± 7.5	24.7 ± 6.6	23.9 ± 8.1	0.752
change	−0.7 ± 6.7	−0.8 ± 6.5	1.1 ± 9.7	1.5 ± 8.0	0.362
Carbohydrate, % total energy					
baseline	58.7 ± 10.4	58.3 ± 12.0	58.3 ± 11.9	59.2 ± 9.6	0.975
week 12	60.0 ± 12.0	58.4 ± 9.7	56.5 ± 9.6	56.8 ± 11.0	0.358
change	1.3 ± 9.2	0.1 ± 10.6	−1.8 ± 14.3	−2.5 ± 11.1	0.376
Total cholesterol, mg/d					
baseline	558.0 ± 342.0	535.9 ± 183.1	579.8 ± 365.8	564.0 ± 301.2	0.921
week 12	502.6 ± 349.7	546.9 ± 199.3	623.1 ± 352.6	543.4 ± 285.8	0.289
change *	−22.1 (−118.3, 24.3)	0 (−71.5, 99.0)	0 (−87.7, 132.4)	10.4 (−132.3, 99.2)	0.174
Food groups, g/d					
Total grains, g/d					
baseline *	481.8 (384.1, 574.8)	480.8 (359.6, 664.2)	499.5 (316.8, 649.2)	478.4 (333.5, 662.7)	0.459
week 12 *	490.0 (382.4, 573.3)	498.8 (302.3, 703.4)	465.2 (328.9, 610.2)	467.0 (286.3, 558.5)	0.953
change	1.4 ± 156.8	6.6 ± 229.9	−20.8 ± 241.7	−56.5 ± 202.0	0.465
Vegetables, g/d					
baseline	600.0 ± 608.8	597.2 ± 266.4	615.0 ± 393.8	595.4 ± 457.9	0.892
week 12	609.3 ± 624.6	620.0 ± 246.9	526.6 ± 251.3	582.6 ± 390.3	0.695
change	−50.7 ± 275.1	22.9 ± 274.2	−88.4 ± 319.8	−12.8 ± 426.4	0.406
Fruits, g/d					
baseline	245.6 ± 273.5	214.8 ± 255.2	222.2 ± 177.3	247.2 ± 350.1	0.918
week 12	192.6 ± 149.8	261.8 ± 239.5	220.1 ± 135.4	245.7 ± 324.4	0.479
change	−52.9 ± 260.8	46.9 ± 254.5	−2.1 ± 202.8	−1.4 ± 211.5	0.244
Red and processed meat, g/d					
baseline	108.9 ± 78.9	96.8 ± 92.4	113.5 ± 100.0	105.5 ± 99.0	0.850
week 12	93.9 ± 68.4	102.4 ± 102.1	108.8 ± 94.7	99.4 ± 69.1	0.864
change	−15.0 ± 72.29	5.6 ± 102.2	−4.7 ± 122.0	−6.1 ± 78.5	0.784
Sedentary time, hrs/d					
baseline	2.348 ± 2.063	2.500 ± 1.880	2.326 ± 1.898	2.576 ± 2.119	0.918
week 12	2.435 ± 2.177	2.526 ± 2.400	2.630 ± 2.339	2.614 ± 2.703	0.979
change	0.087 ± 2.140	0.026 ± 2.111	0.304 ± 1.618	0.038 ± 2.313	0.909
Total PA, Met/h/week					
baseline	87.7 ± 66.7	67.6 ± 62.7	65.6 ± 58.0	67.6 ± 52.4	0.251
week 12 *	62.1 (34.6, 140.9)	44.5 (26.1, 112.0)	49.0 (28.6, 75.3)	49.0 (25.9, 84.5)	0.385
change	−0.741 ± 64.457	6.905 ± 41.632	−6.325 ± 55.652	−4.049 ± 54.275	0.678

Data were presented as mean ± SD with comparison by ANOVA or median (P_25_–P_75_) with comparison by Kruskal–Wallis test if unequal variance was identified. * *p* value by Kruskal–Wallis test. Trend analysis across different dosage groups was conducted by either parametric (ANOVA, linear trend) or non-parametric (Jonckheere-Terpstra) approach when applicable. For dietary total energy intake, 5 cases (3 at baseline and 2 at week 12) had more than 5000 kcal/d. They were replaced with 5000 kcal/d. The macro-nutrients were expressed as percentages of total energy (% total energy). Abbreviations: PA, physical activity; SD, standard deviation; ANOVA, analysis of variance. Energy expenditure for total physical activity were estimated by adding mild, moderate and intensive PA energy expenditures (Met/h/week).

**Table 3:** Effects of different dosages of purified anthocyanins on bilateral brachial-ankle pulse wave velocity (baPWV) and ankle brachial index (ABI) by intention to treat analysis (*n* = 184).

	Supplementation of Purified Anthocyanins					
	Placebo (*n* = 46)	160 mg/d (*n* = 46)	320 mg/d (*n* = 46)	640 mg/d (*n* = 46)	*P*_overall_	*P*_trend_
Right PWV, cm/s						
baseline	1587.3 ± 312.8	1559.8 ± 335.9	1560.3 ± 333.2	1486.6 ± 298.9	0.490	
week 12	1552.6 ± 300.1	1563.8 ± 317.3	1519.8 ± 263.2	1468.7 ± 283.4	0.401	
change	−34.6 ± 193.1	5.26 ± 233.6	−40.4 ± 230.1	−23.5 ± 156.6	0.719	0.514
change%	−1.64 ± 10.85	1.40 ± 14.49	−0.42 ± 18.71	−1.00 ± 10.21	0.751	0.547
Left PWV, cm/s						
baseline	1586.0 ± 314.3	1590.5 ± 352.7	1532.3 ± 326.7	1481.0 ± 287.7	0.325	
week 12	1523.1 ± 287.2	1565.5 ± 339.0	1506.0 ± 287.6	1451.1 ± 273.1	0.325	
change	−62.9 ± 210.9	−24.3 ± 251.1	−26.3 ± 205.0	−30.4 ± 168.6	0.796	0.760
change%	−3.14 ± 12.22	−0.43 ± 14.75	−0.22 ± 14.95	−1.45 ± 10.91	0.711	0.602
Max-PWV, cm/s						
baseline	1620.5 ± 317.4	1601.1 ± 353.9	1580.0 ± 333.3	1510.6 ± 296.5	0.405	
week 12	1575.8 ± 300.6	1605.4 ± 332.3	1550.8 ± 266.4	1492.5 ± 280.5	0.309	
change	−44.7 ± 192.5	5.4 ± 247.6	−29.3 ± 212.8	−18.4 ± 161.3	0.699	0.747
change%	−2.21 ± 10.53	1.53 ± 14.47	0.02 ± 16.64	−0.63 ± 10.17	0.601	0.715
Right ABI						
baseline	1.143 ± 0.067	1.155 ± 0.079	1.153 ± 0.087	1.144 ± 0.082	0.866	0.981
week 12	1.147 ± 0.088	1.153 ± 0.093	1.154 ± 0.082	1.135 ± 0.092	0.713	0.604
change	0.003 ± 0.081	0.004 ± 0.083	0.001 ± 0.072	−0.011 ± 0.082	0.798	0.870
change%	0.419 ± 7.185	0.506 ± 6.894	0.282 ± 6.121	−0.787 ± 7.094	0.785	0.843
Left ABI						
baseline	1.137 ± 0.071	1.158 ± 0.088	1.137 ± 0.086	1.130 ± 0.084	0.420	0.466
week 12	1.132 ± 0.071	1.145 ± 0.092	1.136 ± 0.094	1.126 ± 0.084	0.757	0.618
change	−0.004 ± 0.062	−0.007 ± 0.092	−0.000 ± 0.065	−0.003 ± 0.100	0.985	0.942
change%	−0.237 ± 5.496	−0.274 ± 7.704	0.085 ± 5.811	0.095 ± 9.140	0.991	0.988
Min-ABI						
baseline	1.119 ± 0.069	1.126 ± 0.077	1.120 ± 0.076	1.114 ± 0.077	0.893	
week 12	1.117 ± 0.085	1.123 ± 0.088	1.122 ± 0.084	1.109 ± 0.085	0.858	
change	−0.001 ± 0.082	−0.003 ± 0.069	0.002 ± 0.059	−0.005 ± 0.088	0.976	0.883
change%	0.051 ± 7.311	−0.172 ± 5.952	0.228 ± 5.256	−0.226 ± 8.196	0.988	0.924

The analysis was made by analysis of variance (ANOVA) with results being presented as mean ± SD. Abbreviations: ABI: ankle brachial index. The max-PWV was the higher baPWV at either the left or right side. The min-ABI was estimated by dividing the lower side of the ankle SBP with the higher side of brachial SBP. Change = data at 3-month − data at baseline. Change% = (data at 3-month − data at baseline) × 100/data at baseline.

**Table 4:** Effects of different dosages of purified anthocyanins on brachial and ankle systolic and diastolic blood pressures by intention to treat analysis (*n* = 184).

	Supplementation of Purified Anthocyanins					
	Placebo (*n* = 46)	160 mg/d (*n* = 46)	320 mg/d (*n* = 46)	640 mg/d (*n* = 46)	*P*_overall_	*P*_trend_
Left-brachial SBP, mmHg						
baseline	129.2 ± 13.2	125.7 ± 13.3	127.4 ± 13.3	127.1 ± 19.2	0.693	
week 12	125.1 ± 11.1	122.7 ± 12.8	123.8 ± 11.4	122.0 ± 18.1	0.742	
change	−4.2 ± 10.5	−2.9 ± 11.2	−3.6 ± 10.4	−5.0 ± 10.6	0.749	0.609
change%	−2.8 ± 8.1	−2.0 ± 8.5	−2.4 ± 7.7	−3.6 ± 8.3	0.752	0.572
Left-brachial DBP, mmHg						
baseline	73.8 ± 8.7	71.7 ± 9.5	73.7 ± 9.4	74.1 ± 11.7	0.562	
week 12	71.8 ± 8.0	70.9 ± 9.6	71.9 ± 9.3	72.4 ± 11.2	0.885	
change	−2.00 ± 7.15	−0.89 ± 7.72	−1.78 ± 8.46	−1.70 ± 7.52	0.841	0.965
change%	−2.18 ± 9.65	−0.71 ± 10.45	−1.75 ± 11.88	−1.76 ± 10.12	0.865	0.992
Left-brachial MAP, mmHg						
baseline	97.0 ± 12.2	95.0 ± 11.5	95.7 ± 11.2	95.5 ± 15.5	0.849	
week 12	94.4 ± 10.0	92.0 ± 11.4	93.5 ± 10.1	93.3 ± 15.0	0.828	
change	−2.63 ± 10.23	−3.02 ± 9.33	−2.15 ± 10.17	−2.24 ± 8.27	0.993	0.795
change%	−1.94 ± 10.88	−2.77 ± 9.52	−1.62 ± 10.19	−1.96 ± 8.62	0.986	0.912
Right-brachial SBP, mmHg						
baseline	129.3 ± 13.8	125.4 ± 13.4	125.6 ± 13.9	126.1 ± 19.8	0.561	
week 12	125.3 ± 12.7	123.9 ± 13.3	124.2 ± 11.8	121.5 ± 17.3	0.626	
change	−4.0 ± 11.5	−1.5 ± 10.9	−1.3 ± 11.9	−4.6 ± 9.2	0.297	0.785
change%	−2.7 ± 8.8	−0.8 ± 8.7	−0.5 ± 9.4	−3.0 ± 8.3	0.353	0.862
Right-brachial DBP, mmHg						
baseline	74.4 ± 9.5	71.9 ± 9.5	72.4 ± 9.2	73.0 ± 14.5	0.661	
week 12	71.8 ± 9.2	70.7 ± 9.3	72.1 ± 9.1	71.0 ± 11.7	0.902	
change	−2.6 ± 8.6	−1.2 ± 6.5	−0.3 ± 7.4	−2.1 ± 8.1	0.449	0.670
change%	−2.8 ± 11.9	−1.2 ± 9.3	0.1 ± 10.6	−0.1 ± 24.7	0.789	0.368
Right-brachial MAP, mmHg						
baseline	97.8 ± 12.3	94.9 ± 11.1	94.5 ± 11.9	94.8 ± 17.5	0.579	
week 12	94.6 ± 10.6	93.3 ± 10.8	94.8 ± 10.4	92.6 ± 14.4	0.785	
change	−3.2 ± 10.7	−1.6 ± 8.2	0.3 ± 11.4	−2.1 ± 9.4	0.386	0.485
change%	−2.5 ± 11.4	−1.3 ± 8.9	1.1 ± 12.1	−0.6 ± 16.2	0.573	0.355
Left-ankle SBP, mmHg						
baseline	149.5 ± 20.3	146.8 ± 18.1	146.7 ± 21.0	145.9 ± 26.3	0.872	
week 12	144.4 ± 16.8	143.8 ± 17.3	143.4 ± 17.9	139.6 ± 23.0	0.621	
Change	−5.1 ± 15.2	−3.0 ± 14.1	−3.3 ± 12.4	−6.3 ± 16.1	0.665	0.685
change%	−2.7 ± 9.7	−1.6 ± 9.6	−1.7 ± 7.9	−3.5 ± 10.7	0.739	0.693
Right-ankle SBP, mmHg						
baseline	150.4 ± 19.1	146.5 ± 18.3	148.8 ± 20.8	147.9 ± 25.4	0.851	
week 12	146.0 ± 17.7	144.5 ± 16.7	145.8 ± 18.4	140.7 ± 23.4	0.525	
Change	−4.3 ± 14.8	−2.0 ± 12.9	−3.0 ± 14.2	−7.1 ± 13.4	0.325	0.307
change%	−2.4 ± 9.4	−0.9 ± 8.5	−1.4 ± 9.6	−4.4 ± 8.6	0.276	0.276
sIAD, mmHg						
baseline	4.130 ± 3.110	4.370 ± 4.255	4.935 ± 4.459	4.348 ± 5.551	0.842	
week 12	4.326 ± 3.590	4.544 ± 4.722	4.544 ± 5.184	4.239 ± 4.100	0.983	
Change	0.196 ± 4.612	0.174 ± 2.719	−0.391 ± 4.674	−0.109 ± 7.024	0.935	0.654
change% *	0 (−60.0, 100.0)	0 (−50.0, 76.7)	0 (−60.0, 50.0)	0 (−58.3, 200.0)	0.479	0.710
sIAND, mmHg						
baseline	5.500 ± 3.494	6.174 ± 6.819	6.283 ± 6.473	6.044 ± 4.477	0.907	
week 12	5.783 ± 6.401	6.609 ± 6.923	5.739 ± 5.277	5.196 ± 5.171	0.728	
change	0.283 ± 7.638	0.435 ± 7.562	−0.544 ± 6.706	−0.848 ± 6.606	0.788	0.355
change%	80.4 ± 356.2	55.2 ± 272.7	74.7 ± 288.2	38.7 ± 188.9	0.896	0.872

The analysis was made by analysis of variance (ANOVA) with results being presented as mean ± SD. * Results of heterogenous variance were presented as median (inter-quartile range P_25_–P_75_) and compared by Kruskal–Wallis ANOVA with trend analysis by Jonckheere–Terpstra approach. Abbreviations: sIAD, systolic inter-arm difference; sIAND, systolic inter-ankle difference; SBP, systolic blood pressure; DBP, diastolic blood pressure; MAP, mean arterial blood pressure. Change = final value − baseline value; change% = (final value − baseline value) × 100%/baseline value.

**Table 5:** Effects of different dosages of purified anthocyanins on composite cardiovascular variables by intention to treat analysis, (*n* = 184).

	Supplementation of Purified Anthocyanins					
	Placebo (*n* = 46)	160 mg/d (*n* = 46)	320 mg/d (*n* = 46)	640 mg/d (*n* = 46)	*P*_overall_	*P*_trend_
RC, mmol/L						
baseline	0.599 ± 0.522	0.476 ± 0.328	0.650 ± 0.527	0.615 ± 0.560	0.367	
week 12	0.564 ± 0.438	0.541 ± 0.388	0.556 ± 0.465	0.652 ± 0.642	0.705	
change	−0.035 ± 0.374	0.064 ± 0.273	−0.094 ± 0.243	0.037 ± 0.271	0.048	0.773
change% *	2.7 (−31.0, 39.9)	0 (−18.4, 47.8)	−6.8 (−32.2, 10.8)	13.2 (−17.6, 75.1)	0.034	0.746
Non-HDL-c, mmol/L						
baseline	4.000 ± 1.001	3.764 ± 1.067	3.899 ± 1.081	3.926 ± 0.964	0.742	
week 12	3.933 ± 0.965	3.992 ± 1.065	3.944 ± 1.084	3.905 ± 0.988	0.982	
change	−0.065 ± 0.520	0.228 ± 0.570	0.044 ± 0.489	−0.021 ± 0.468	0.037	0.877
change%	−0.340 ± 13.471	7.371 ± 16.567	1.747 ± 13.070	0.128 ± 12.340	0.033	0.647
TG/HDL-c						
baseline	1.609 ± 2.256	1.081 ± 0.851	1.616 ± 1.406	1.814 ± 3.120	0.379	
week 12	1.341 ± 1.320	1.187 ± 1.485	1.333 ± 0.892	1.686 ± 3.312	0.666	
change	−0.269 ± 1.316	0.106 ± 0.854	−0.283 ± 1.005	−0.128 ± 1.346	0.338	0.965
change%	7.381 ± 56.469	7.292 ± 44.389	−4.429 ± 32.179	−0.310 ± 30.692	0.451	0.213
TyG-index						
baseline	8.989 ± 0.548	8.779 ± 0.475	9.044 ± 0.662	8.976 ± 0.626	0.144	
week 12	8.988 ± 0.498	8.816 ± 0.552	8.966 ± 0.560	8.930 ± 0.595	0.452	
change	−0.001 ± 0.378	0.038 ± 0.325	−0.078 ± 0.346	−0.046 ± 0.302	0.382	0.262
change%	0.100 ± 4.185	0.447 ± 3.700	−0.728 ± 3.651	−0.439 ± 3.366	0.433	0.259
TyG-BMI						
baseline	225.7 ± 29.1	210.3 ± 31.0	221.3 ± 33.7	212.6 ± 39.4	0.097	
week 12	225.7 ± 32.8	211.5 ± 32.0	219.1 ± 30.9	210.9 ± 39.8	0.126	
change	−0.047 ± 12.368	1.200 ± 9.679	−2.238 ± 11.054	−1.712 ± 9.066	0.390	0.230
change%	−0.098 ± 5.426	0.627 ± 4.655	−0.704 ± 4.825	−0.739 ± 4.222	0.484	0.306
VAI						
baseline *	1.608 (1.151, 2.385)	1.318 (0.878, 2.086)	1.731 (1.209, 3.516)	1.425 (1.065, 2.477)	0.206	
week 12	2.400 ± 2.418	1.876 ± 2.036	2.186 ± 1.442	2.998 ± 6.596	0.533	
change	−0.454 ± 2.411	0.095 ± 1.198	−0.530 ± 1.760	−0.292 ± 2.504	0.473	0.919
change%	8.504 ± 57.948	6.017 ± 44.244	−6.004 ± 31.911	−2.144 ± 30.090	0.321	0.119
FRS						
baseline	14.4 ± 3.3	13.5 ± 4.1	14.0 ± 3.7	13.2 ± 4.4	0.482	
week 12	14.1 ± 3.4	13.9 ± 4.1	13.9 ± 3.9	13.0 ± 4.2	0.507	
change	−0.3 ± 1.7	0.3 ± 1.8	−0.04 ± 1.5	−0.2 ± 2.0	0.359	0.953
change%	−1.7 ± 13.4	5.9 ± 26.2	−0.2 ± 11.1	4.0 ± 31.9	0.335	0.460

Data were presented as mean ± SD with comparison by ANOVA. * If unequal variance was identified, data were presented as median (P_25_–P_75_) with group comparison by Kruskal–Wallis and trend analysis by Jonckheere–Terpstra approach. Formulas: RC = TC-HDL-c-LDL-c; Non-HDL-c = LDL-c + 1/5 × TG; TyG-index = In(TG(mg/dL) × FG(mg/dL)/2); TyG-BMI = TyG-index × BMI; Visceral adiposity index (VAI) = WC/(39.68 + 1.88 × BMI) × (TG/1.03) × (1.31/HDL-c) (for males); and WC/(36.58 + 1.89 × BMI) × (TG/0.81) × (1.52/HDL-c) for females. Change = final value − baseline value; change% = (final value − baseline value) × 100%/baseline value. Abbreviations: RC, remnant cholesterol; TG, triglycerides; TC, total cholesterol; TyG-index, triglycerides-glucose index; TyG-BMI, triglycerides-glucose body mass index; Non-HDL-c, non-high density lipoprotein cholesterol; VAI, visceral adiposity index; FRS: Framingham risk score; WC, waist circumference.

## Data Availability

The original datasets are not publicly available due to ethical limitations on publishing medical record data, but are available from the corresponding author on reasonable request under a strict confidential process.

## Supplementary Materials

The following supporting information can be downloaded at: https://www.mdpi.com/article/10.3390/nu18010112/s1. Reference [58] is cited in the supplementary materials. **Table S1.** Per-protocol analysis for the effects (change and change%) of anthocyanins on arterial stiffness, four-limb blood pressures and composite cardiovascular markers among participants who had good compliance by multivariable general linear model (*n* = 161); **Table S2.** Sensitivity analyses by linear mixed model (LMM) with individual participants as random factor and adjustment for baseline data on the week-12 change and change% of arterial stiffness, four-limb blood pressures and composite cardiovascular markers (*n* = 184); **Table S3.** Sensitivity analyses by exclusion of patients under medications for lowering glucose, lipids, or thyroid conditions for the effects of anthocyanins (3-month change and change%) on arterial stiffness, four-limb blood pressures and composite cardiovascular markers by ANCOVA; **Table S4.** Subgroup analyses by gender (female and male) for the effects of anthocyanins on the pre-post change% of arterial stiffness, four-limb blood pressure and composite cardiovascular markers by ANCOVA; **Table S5.** Subgroup analyses by age (<65 and ≥65 yrs) for the effects of anthocyanins on the pre-post change% of arterial stiffness, four-limb blood pressure and composite cardiovascular markers by ANCOVA; **Table S6.** Subgroup analyses by body mass index (BMI <24 vs. ≥24 kg/m^2^) for the effects of anthocyanins on the pre-post change% of arterial stiffness, four-limb blood pressures and composite cardiovascular markers by ANCOVA; **Table S7.** Subgroup analyses by severity of hyperglycemia (pre-diabetes vs. diabetes) for the effects of anthocyanins on the pre-post change% of arterial stiffness, four-limb blood pressures and composite cardiovascular markers by ANCOVA; **Table S8.** Subgroup analyses by baseline hypertension for the effects of anthocyanins on the pre-post change% of arterial stiffness, four-limb blood pressure and composite cardiovascular markers by ANCOVA; **Table S9.** Subgroup analyses by baseline hyperlipidemia for the effects of anthocyanins on the pre-post change% of arterial stiffness, four-limb blood pressures and composite cardiovascular markers by ANCOVA; **Table S10.** CONSORT 2025 checklist item description; **Figure S1.** Max-PWV change and change% by different dosages of ACNs; **Figure S2.** Min-ABI change and change% by different dosages of ACNs; **Figure S3.** Left brachial SBP change and change% by different dosages of ACNs; **Figure S4.** Left brachial DBP change and change% by different dosages of ACNs; **Figure S5.** sIAD change and change% by different dosages of ACNs; **Figure S6.** sIAND change and change% by different dosages of ACNs; **Figure S7.** RC change and change% by different dosages of ACNs; **Figure S8.** Non-HDL change and change% by different dosages of ACNs; **Figure S9.** TG/HDL-c change and change% by different dosages of ACNs; **Figure S10.** TyG-BMI change and change% by different dosages of ACNs; **Figure S11.** VAI change and change% by different dosages of ACNs; **Figure S12.** FRS change and change% by different dosages of ACN; **Figure S13.** TyG-index change and change% by different dosages of ACN.
