# Supplementary material for: Purified Anthocyanins Indicated No Significant Effect on Arterial Stiffness, Four-Limb Blood Pressures and Cardiovascular Risk—A 12-Week Dose–Response Trial in Chinese Middle-Aged and Elderly Adults with Hyperglycemia
Source: Nutrients. 2025 Dec 29;18(1):112. doi: 10.3390/nu18010112 (PMC12788160; doi:10.3390/nu18010112)
Supplement: Supplementary file 1 [file nutrients-18-00112-s001.zip › Supplementary Figures and Tables.pdf]

Supplemental Figures and Tables

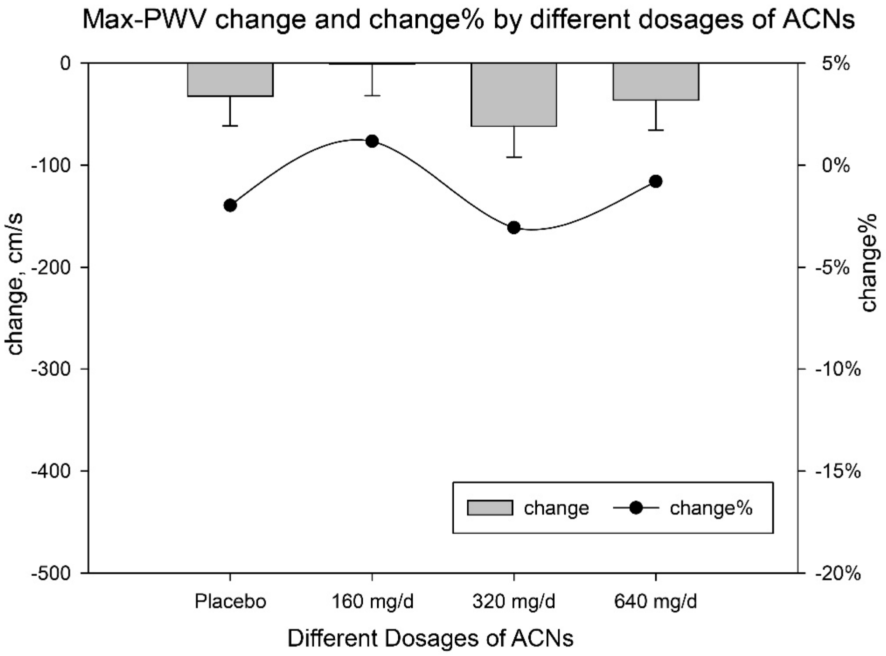

Figure S1. Max-PWV change and change% by different dosages of ACNs

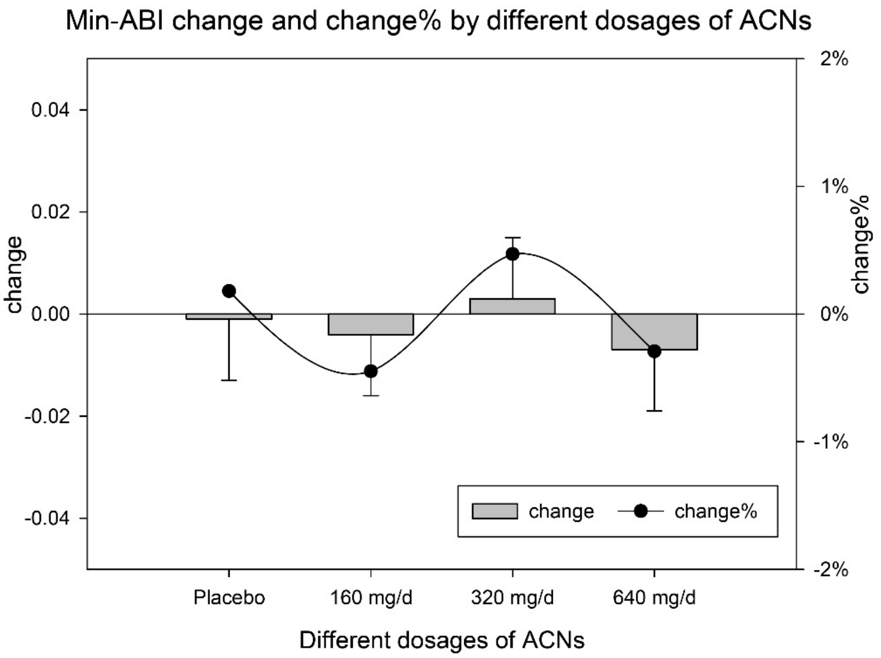

Figure S2. Min-ABI change and change% by different dosages of ACNs

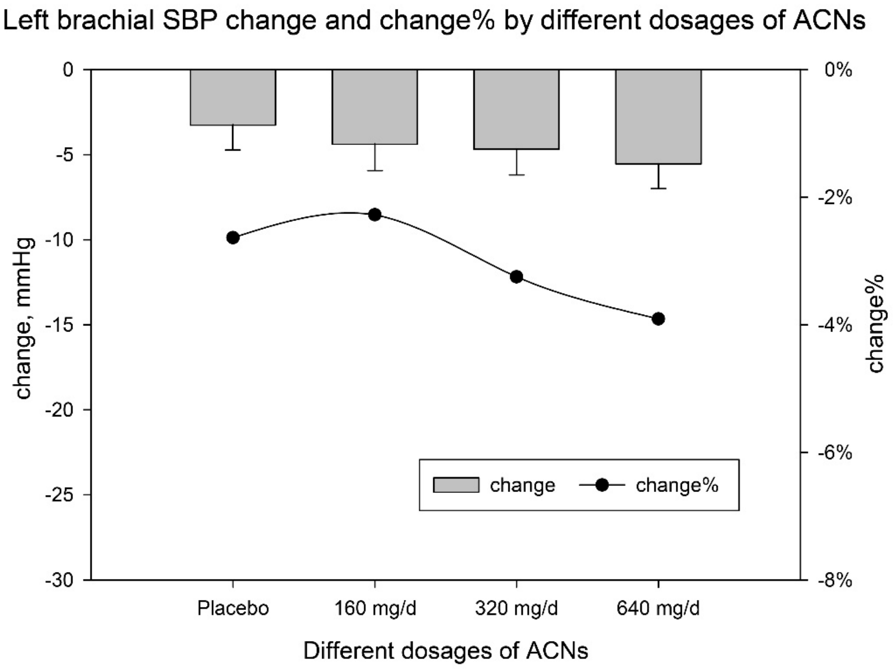

Figure S3. Left brachial SBP change and change% by different dosages of ACNs

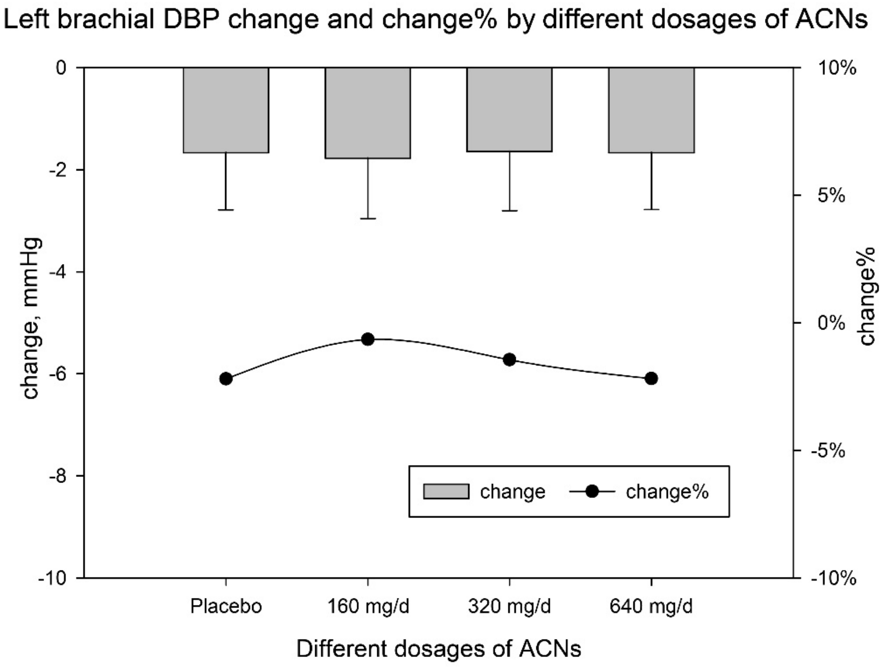

Figure S4. Left brachial DBP change and change% by different dosages of ACNs

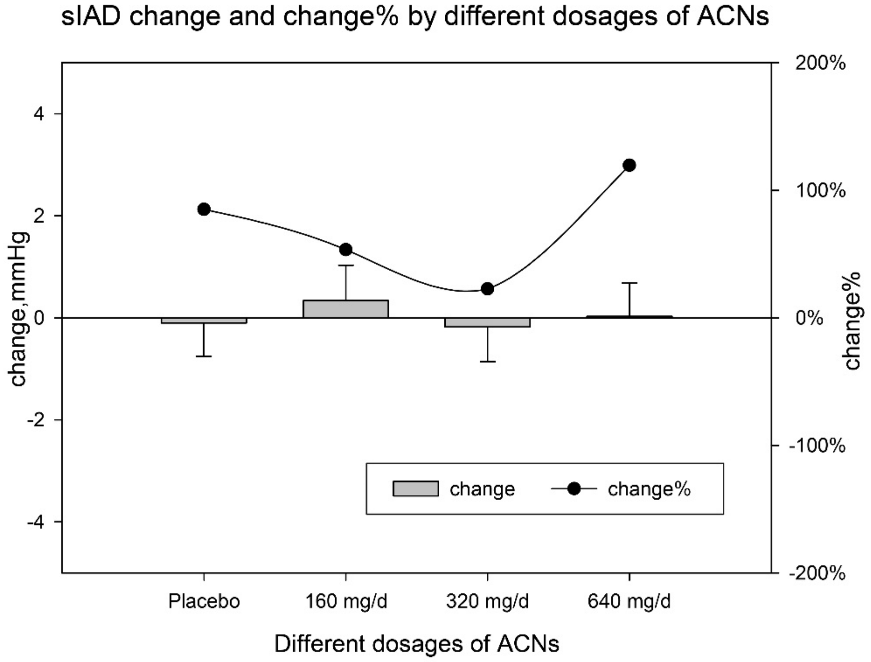

Figure S5. sIAD change and change% by different dosages of ACNs

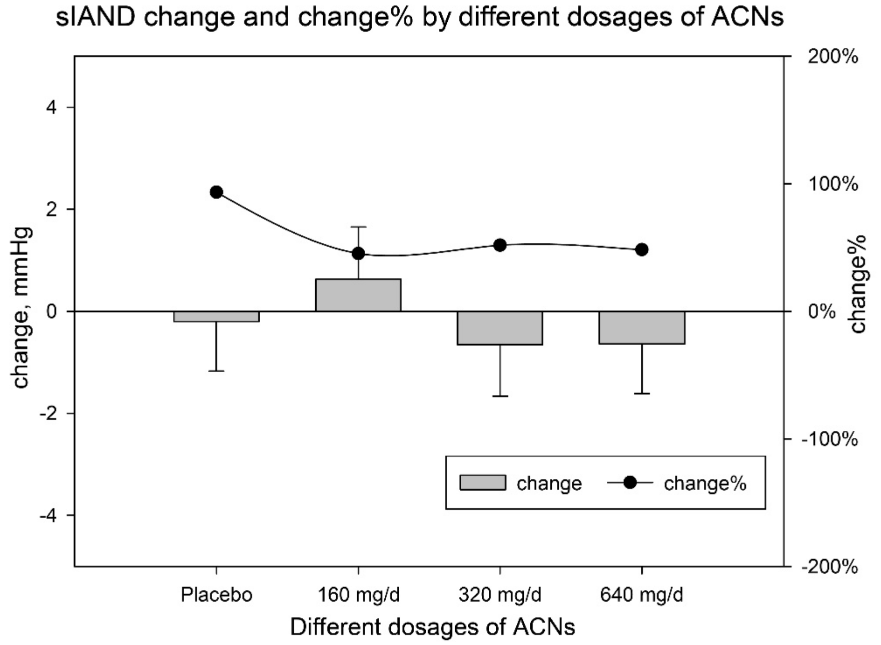

Figure S6. sIAND change and change% by different dosages of ACNs

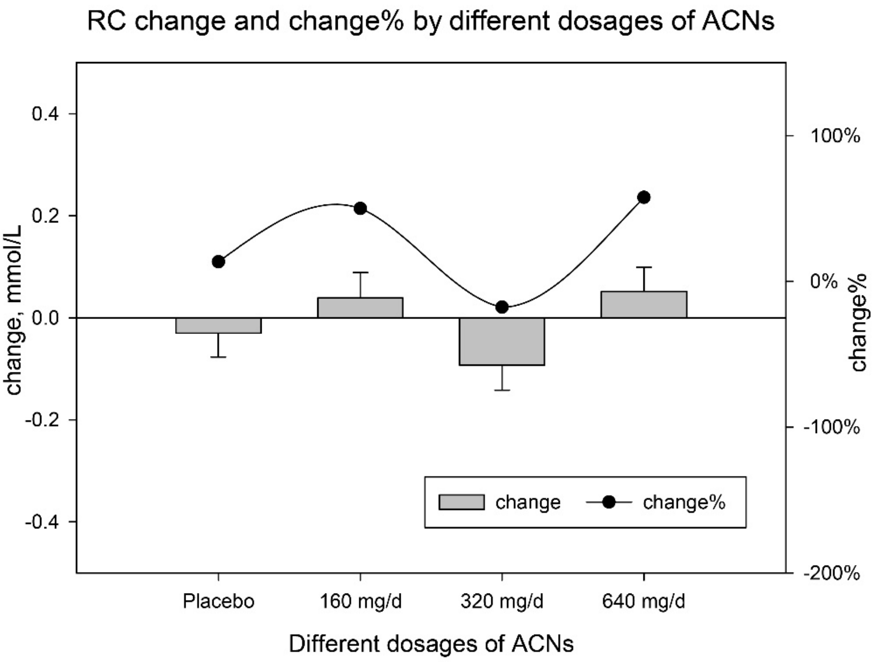

Figure S7. RC change and change% by different dosages of ACNs

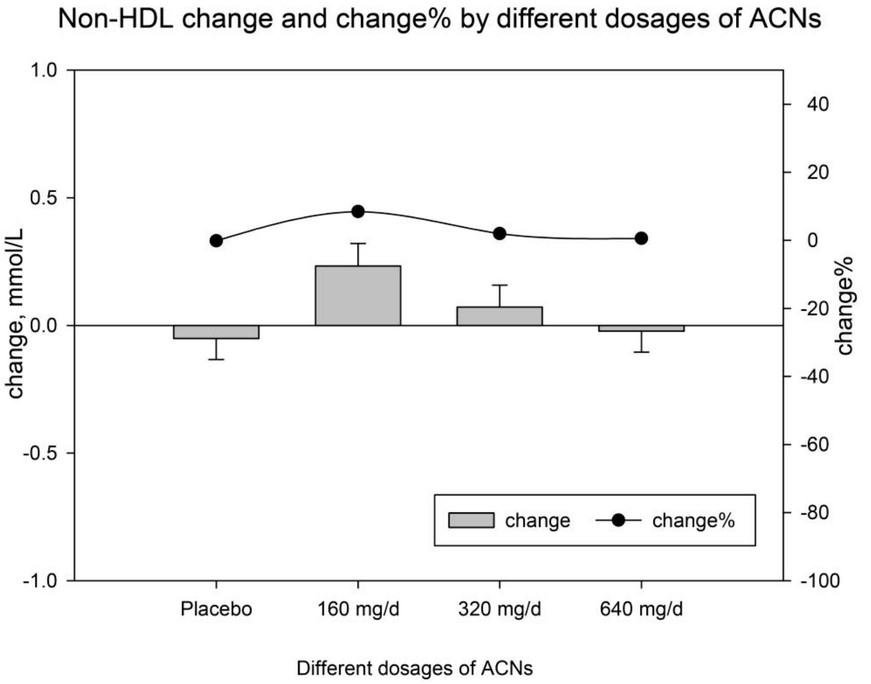

Figure S8. Non-HDL change and change% by different dosages of ACNs

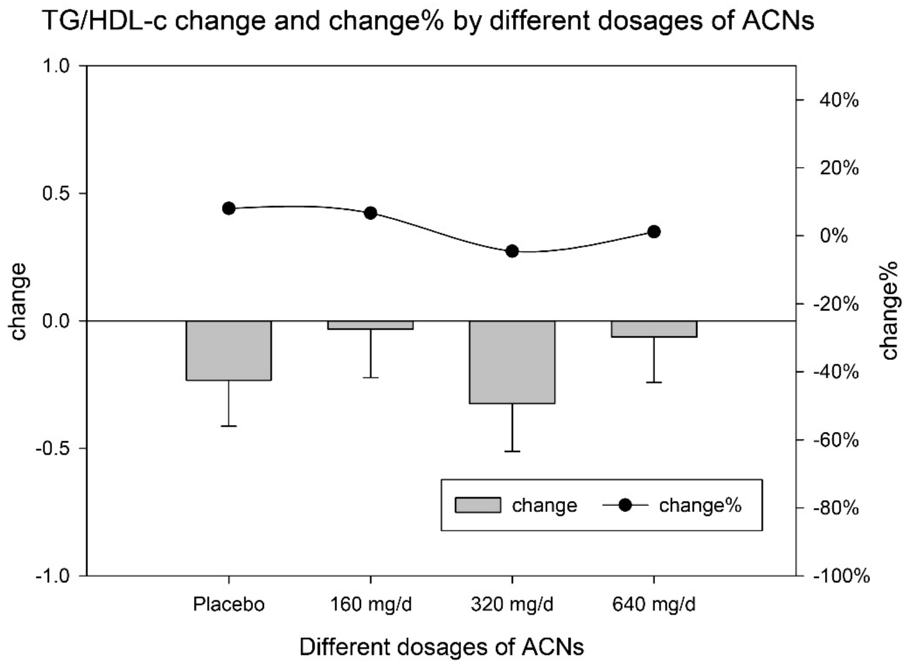

Figure S9. TG/HDL-c change and change% by different dosages of ACNs

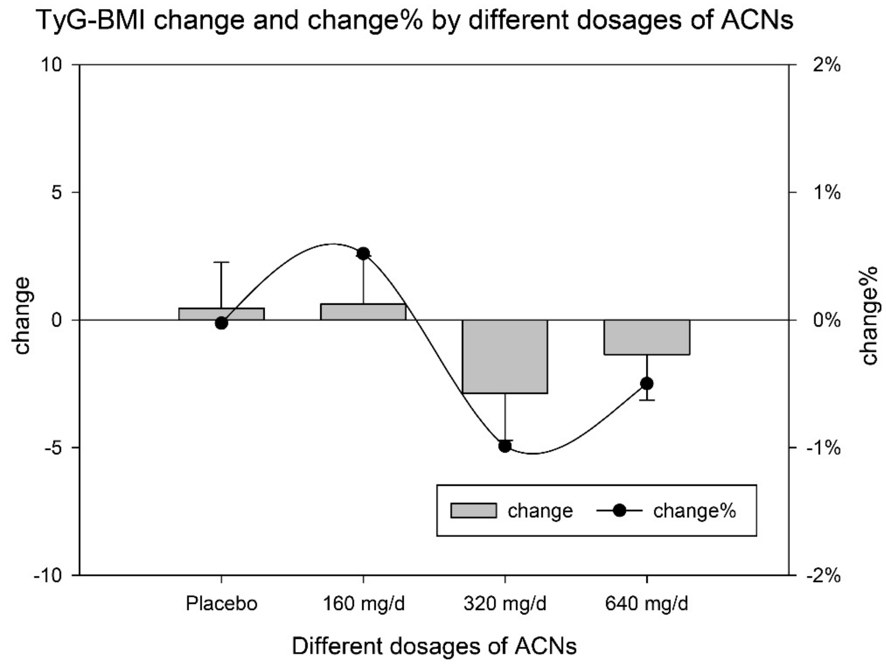

Figure S10. TyG-BMI change and change% by different dosages of ACNs

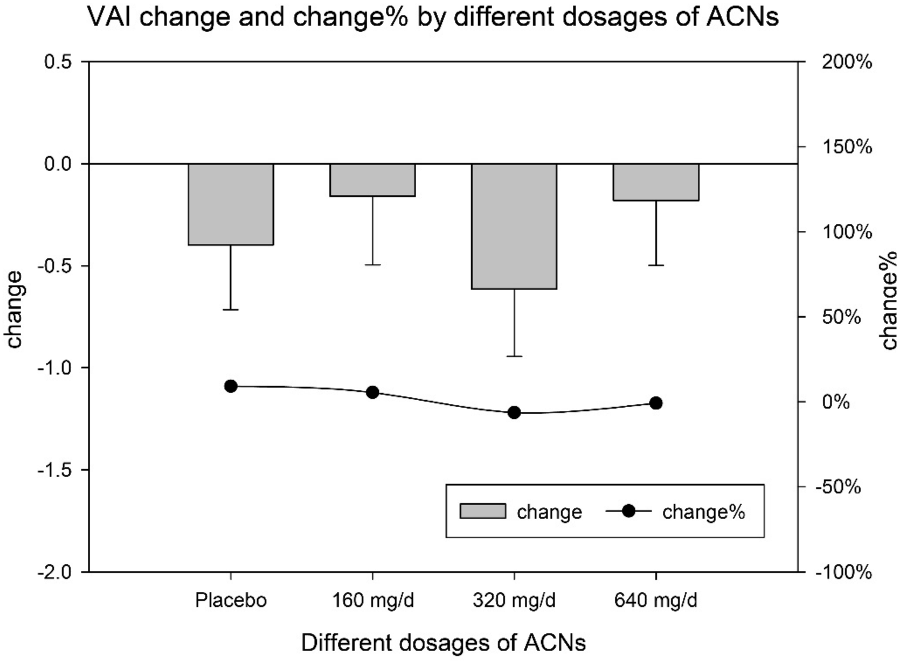

Figure S11. VAI change and change% by different dosages of ACNs

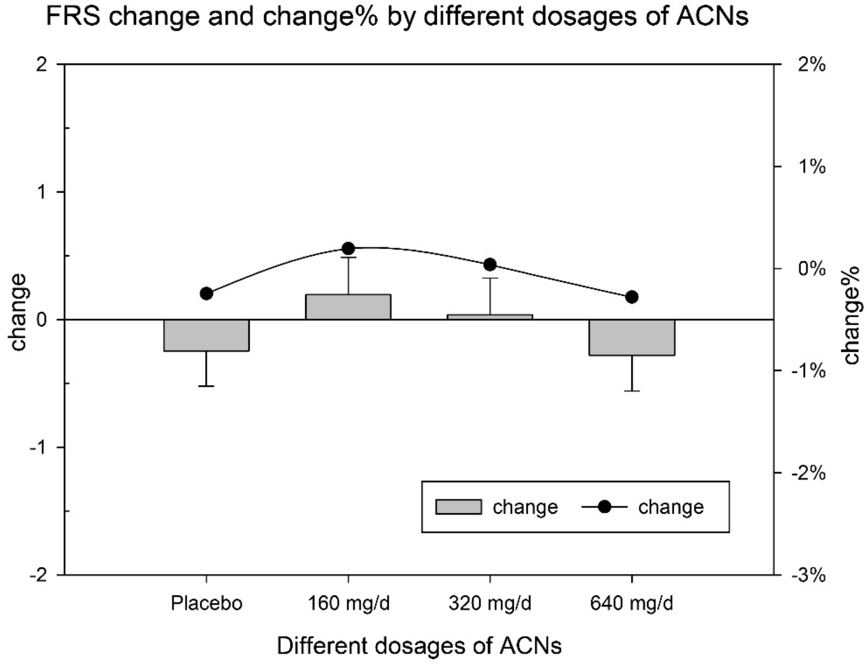

Figure S12. FRS change and change% by different dosages of ACN

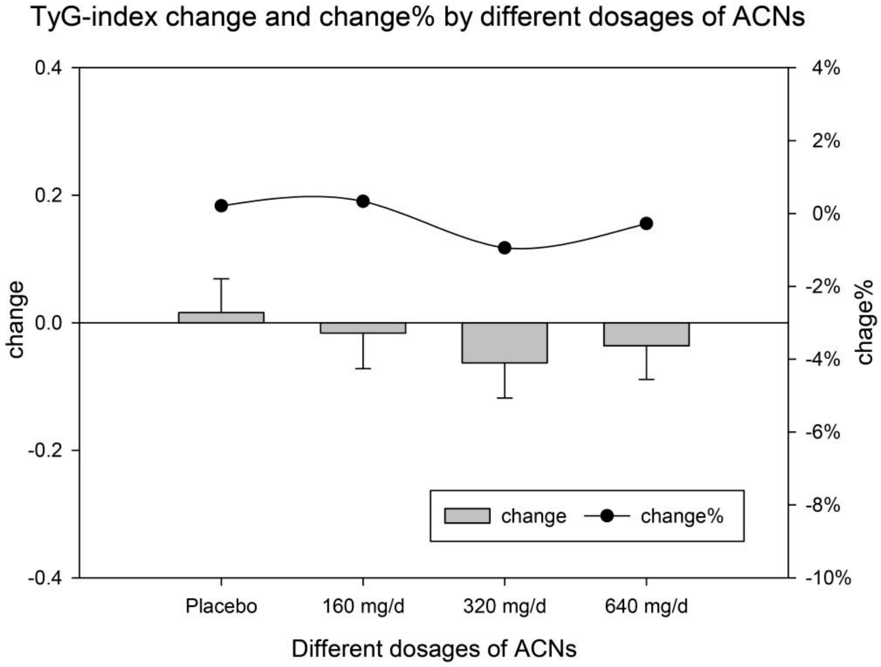

Figure S13. TyG-index change and change% by different dosages of ACN

Table S1. Per-protocol analysis for the effects (change and change%) of anthocyanins on arterial stiffness, four-limb blood pressures and composite cardiovascular markers among participants who had good compliance by multivariable general linear model (n=161)

	Anthocyanins supplementation					
	Placebo	160 mg/d	320 mg/d	640 mg/d	$P_{overall}$	P_{trend}
n	42	38	39	42		
**Max PWV, cm/s**						
change	-32.234±29.238	-0.738±31.087	-61.676±30.338	-36.061±29.551	0.574	0.889
change%	-1.976±1.873	1.174±1.993	-3.065±1.945	-0.798±1.889	0.461	0.951
**Min ABI**						
change	-0.001±0.012	-0.004±0.012	0.003±0.012	-0.007±0.012	0.949	0.886
change%	0.180±1.146	-0.449±1.206	0.470±1.189	-0.294±1.143	0.942	0.918
**Left brachial SBP**						
change	-3.230 ± 1.490	-4.360 ± 1.569	-4.647 ± 1.542	-5.510 ± 1.482	0.751	0.581
change%	-2.633 ± 1.349	-2.275 ± 1.419	-3.247 ± 1.399	-3.907 ± 1.345	0.847	0.542
**Left brachial DBP**						
change	-1.657 ± 1.126	-1.765 ± 1.191	-1.634 ± 1.167	-1.657 ± 1.123	1.000	0.960
change%	-2.204 ± 1.751	-0.648 ± 1.842	-1.453 ± 1.817	-2.191 ± 1.746	0.923	0.985
**IAD of SBP, mmHg**						
change	-0.099 ± 0.657	0.339 ± 0.691	-0.179 ± 0.682	0.029 ± 0.655	0.953	0.738
change%	85.060 ± 34.279	53.435 ± 37.249	22.663 ± 35.999	119.581 ± 36.661	0.289	0.708
**IAND of SBP, mmHg**						
change	-0.199 ± 0.974	0.628 ± 1.025	-0.654 ± 1.010	-0.643 ± 0.971	0.788	0.374
change%	93.329 ± 45.440	45.313 ± 50.246	51.834 ± 49.773	48.271 ± 45.315	0.871	0.709
**RC, mmol/L**						

change	-0.030 ± 0.047	0.039 ± 0.050	-0.093 ± 0.049	0.052 ± 0.047	0.130	0.786
change%*	7.175(-31.984,47.222)	7.905(-26.993,59.312)	-11.290(-33.458,13.928)	13.961(-17.560,77.887)	0.026	0.537
**Non-HDL, mmol/L**						
change	-0.051 ± 0.083	0.233 ± 0.088	0.072 ± 0.086	-0.022 ± 0.083	0.099	0.881
change%	-0.149 ± 2.317	8.429 ± 2.437	1.985 ± 2.403	0.554 ± 2.310	0.055	0.640
**TG/HDL-c**						
change	-0.233 ± 0.180	-0.033 ± 0.190	-0.325 ± 0.187	-0.063 ± 0.179	0.635	0.995
change%	8.012 ± 7.138	6.675 ± 7.510	-4.544 ± 7.406	1.150 ± 7.118	0.611	0.221
**TyG-index**						
change	0.016 ± 0.053	-0.016 ± 0.056	-0.063 ± 0.055	-0.036 ± 0.053	0.772	0.272
change%	0.210 ± 0.634	0.333 ± 0.667	-0.944 ± 0.657	-0.277 ± 0.632	0.508	0.270
**TyG-BMI**						
change	0.462 ± 1.803	0.629 ± 1.884	-2.868 ± 1.848	-1.355 ± 1.786	0.488	0.254
change%	-0.025 ± 0.804	0.521 ± 0.846	-0.989 ± 0.834	-0.499 ± 0.802	0.617	0.340
**VAI**						
change	-0.397 ± 0.318	-0.159 ± 0.336	-0.614 ± 0.331	-0.180 ± 0.318	0.734	0.896
change%	9.101 ± 7.166	5.431 ± 7.539	-6.401 ± 7.434	-0.906 ± 7.146	0.459	0.123
**FRS**						
change	-0.246 ± 0.277	0.195 ± 0.293	0.037 ± 0.288	-0.280 ± 0.280	0.610	0.982
change%	-1.752 ± 3.642	8.040 ± 3.833	-1.206 ± 3.781	2.827 ± 3.681	0.230	0.582

Data analyses were conducted by multivariable general linear models (GLM/ANCOVA) and presented as estimated mean±standard error. The adjusted covariates included age (yrs), gender (female/male), alcohol drinking (yes/no), medical history of cardiovascular diseases (yes/no), medications for hypertension (yes/no), medications for thyroid conditions (yes/no), pre-post changes of energy-adjusted cholesterol intake (mg/d) and baseline value of the tested variable for change (this adjustment was not made for change%). Post-hoc comparisons were made by LSD approach if the overall p for ANCOVA was significant (<0.05). *p value by Kruskal-Wallis test. Trend analysis across different dosage groups was

conducted by either parametric (ANOVA, linear trend) or non-parametric (Jonckheere-Terpstra) approach when applicable. Abbreviations: PWV, pulse wave velocity; ABI, ankle brachial index; IAD, systolic inter-arm difference; IAND, inter-ankle difference; SBP, systolic blood pressure; DBP, diastolic blood pressure; RC, remnant cholesterol; TG, triglycerides; TyG-index, triglyceride-glucose index; BMI, body mass index; VAI, visceral adiposity index; FRS, Framingham risk score.

Change=final value-baseline value; change%=(final value-baseline value)*100%/baseline value. The maximum PWV was determined by the higher either left or right PWV. The minimal ABI was estimated by dividing the lower one of ankle SBP with the higher one of brachial SBP. RC = TC-HDL-LDL; Non-HDL= LDL+1/5*TG; TyG-index=In(TG(mg/dl)*FG(mg/dl)/2); TyG-BMI=TyG-index*BMI; Visceral adiposity index (VAI)= WC/(39.68+1.88*BMI)*(TG/1.03)*(1.31/HDL-c) (for males), and WC/(36.58+1.89*BMI)*(TG/0.81)*(1.52/HDL-c) for female.

Table S2. Sensitivity analyses by linear mixed model (LMM) with individual participants as random factor and adjustment for baseline data on the week-12 change and change% of arterial stiffness, four-limb blood pressures and composite cardiovascular markers (n=184)

	Anthocyanins supplementation				
	Placebo	160 mg/d	320 mg/d	640 mg/d	$P_{overall}$
n	46	46	46	46	
**Max-PWV, cm/s**					
change	-33.289±27.278	7.175±27.859	-29.143±27.222	-37.773±27.679	0.649
change%	-1.529±1.803	1.896±1.820	0.046±1.799	-1.715±1.829	0.467
**Min-ABI**					
change	-0.002±0.010	-0.001±0.011	0.002±0.010	-0.007±0.010	0.845
change%	0.012±0.938	0.037±0.939	0.232±0.938	-0.401±0.939	0.971
**Left brachial SBP**					
change	-3.507±1.391	-3.240±1.406	-3.575±1.388	-5.097±1.388	0.788
change%	-2.336±1.088	-2.358±1.087	-2.396±1.086	-3.628±1.086	0.797
**Left brachial DBP**					
change	-1.818±1.033	-1.240±1.049	-1.658±1.033	-1.420±1.034	0.981
change%	-1.970±1.427	-1.398±1.432	-1.613±1.427	-1.422±1.428	0.991
**IAD of SBP, mmHg**					
change	-0.010±0.612	0.210±0.618	-0.086±0.612	-0.176±0.611	0.975
change%	73.851±29.420	38.887±29.705	27.429±30.103	105.985±30.772	0.252

**IAND of SBP, mmHg**					
change	-0.108±0.865	0.495±0.874	-0.348±0.864	-0.832±0.864	0.752
change%	67.872±40.467	63.177±42.318	84.564±42.343	34.917±40.361	0.860
**RC, mmol/L**					
change	-0.007±0.001	-0.007±0.001	-0.007±0.001	-0.008±0.001	0.943
change%	8.592±15.997	43.963±16.453	-7.017±16.228	45.881±15.823	0.051
**Non-HDL, mmol/L**					
change	-0.050±0.074	0.200±0.074	0.046±0.073	-0.015±0.073	0.088
change%	0.026±1.991	6.889±1.993[a]	1.756±1.988	0.235±1.988	0.055
**TG/HDL-c**					
change	-0.254±0.158	0.017±0.161	-0.267±0.158	-0.072±0.159	0.517
change%	7.702±6.119	5.473±6.153	-4.081±6.119	0.840±6.132	0.537
**TyG-index**					
change	0.008±0.047	0.001±0.048	-0.027±0.047	-0.040±0.047	0.717
change%	0.198±0.515	0.056±0.521	-0.503±0.517	-0.371±0.515	0.739
**TyG-BMI**					
change	0.230±1.567	0.871±1.590	-2.113±1.567	-1.887±1.569	0.437
change%	0.045±0.710	0.502±0.709	-0.638±0.706	-0.823±0.707	0.519
**VAI**					
change	-0.417±0.279	-0.078±0.284	-0.520±0.279	-0.170±0.280	0.653
change%	8.917±6.152	4.402±6.189	-5.881±6.151	-0.825±6.170	0.357

**FRS**					
change	-0.233±0.253	0.308±0.255	-0.019±0.252	-0.229±0.256	0.395
change%	13.307±7.512	14.571±7.573	6.472±7.490	0.076±7.596	0.504

Data were analyzed by linear mixed model (LMM) with individual patients as random factor and baseline data as covariate. The results for each group were presented as estimated means ± standard error. Abbreviations: PWV, pulse wave velocity; ABI, ankle brachial index; IAD, systolic inter-arm difference; IAND, inter-ankle difference; SBP, systolic blood pressure; DBP, diastolic blood pressure; RC, remnant cholesterol; TyG-index, triglyceride-glucose index; BMI, body mass index; VAI, visceral adiposity index; FRS, Framingham risk score. The maximum PWV was determined by the higher either left or right PWV. The minimal ABI was estimated by dividing the lower one of ankle SBP with the higher one of brachial SBP. RC = TC-HDL-LDL; Non-HDL= LDL+1/5*TG; TyG-index=In(TG(mg/dl)*FG(mg/dl)/2); TyG-BMI=TyG-index*BMI; Visceral adiposity index (VAI)= WC/(39.68+1.88*BMI)*(TG/1.03)*(1.31/HDL-c) (for males), and WC/(36.58+1.89*BMI)*(TG/0.81)*(1.52/HDL-c) for female.

Table S3. Sensitivity analyses by exclusion of patients under medications for lowering glucose, lipids, or thyroid conditions for the effects of anthocyanins (3-month change and change%) on arterial stiffness, four-limb blood pressures and composite cardiovascular markers by ANCOVA

	Anthocyanins supplementation					
	Placebo	160mg/d	320mg/d	640mg/d	$P_{overall}$	P_{trend}
n	38	36	37	33		
**Max PWV, cm/s**						
change	-33.897 ± 32.077	18.422 ± 32.757	-20.149 ± 31.727	-18.318 ± 34.425	0.711	0.918
change%	-2.761 ± 2.363	2.952 ± 2.419	1.182 ± 2.342	-0.355 ± 2.544	0.402	0.876
**Min ABI**						
change	0.001±0.011	-0.004±0.011	0.010±0.011	-0.025±0.012	0.160	0.167
change%	0.573±1.037	-0.538±1.050	1.181±1.028	-2.246±1.104	0.129	0.173
**Left brachial SBP**						
change	-3.546±1.508	-3.003±1.535	-3.471±1.493	-3.233±1.610	0.994	0.835
change%	-2.730±1.268	-1.310±1.284	-2.269±1.257	-2.859±1.350	0.836	0.924
**Left brachial DBP**						
change	-1.186±1.156	-1.026±1.183	-1.242±1.146	-0.669±1.240	0.987	0.925
change%	-1.207±1.754	0.333±1.776	-1.083±1.738	-1.588±1.867	0.885	0.928
**IAD of SBP, mmHg**						
change	-0.117±0.708	0.584±0.717	0.272±0.704	0.860±0.753	0.807	0.733
change%	83.746±38.794	77.912±40.212	17.090±39.618	146.461±42.929	0.189	0.454
**IAND of SBP, mmHg**						
change	-0.779±0.932	1.104±0.944	-0.751±0.923	0.202±0.991	0.444	0.868
change%	47.472±41.734	64.186±44.642	25.704±43.222	92.343±43.989	0.749	0.767
**RC, mmol/L**						

change	-0.062±0.048	0.026±0.050	-0.088±0.048	0.093±0.052	0.048	0.394
change%*	2.703(-30.110,35.208)	3.810(-17.023,54.682)	-2.381(-29.374,13.930)	15.909(-16.667,85.185)	0.030	0.540
**Non-HDL, mmol/L**						
change	-0.084±0.082	0.214±0.083	0.044±0.081	0.066±0.087	0.102	0.715
change%	-0.968±2.273	7.165±2.302	2.083±2.253	2.237±2.420	0.106	0.945
**TG/HDL-c**						
change	-0.230±0.199	-0.025±0.203	-0.364±0.198	0.003±0.214	0.534	0.855
change%	0.491±6.282	6.185±6.363	-6.677±6.226	4.407±6.689	0.488	0.748
**TyG-index**						
change	-0.032±0.049	-0.016±0.050	-0.085±0.049	0.027±0.053	0.484	0.674
change%	-0.247±0.599	0.312±0.607	-0.961±0.594	0.100±0.638	0.465	0.686
**TyG-BMI**						
change	-0.514±1.595	0.046±1.629	-1.948±1.584	0.374±1.703	0.753	0.500
change%	-0.283±0.762	0.559±0.772	-0.716±0.756	-0.188±0.812	0.697	0.577
**VAI**						
change	-0.374±0.352	-0.163±0.357	-0.683±0.348	-0.073±0.378	0.635	0.749
change%	1.174±6.255	5.148±6.336	-8.075±6.661	2.490±6.661	0.471	0.541
**FRS**						
change	-0.366±0.281	0.424±0.291	-0.150±0.278	0.113±0.303	0.261	0.432
change%	-2.398±3.958	9.587±4.082	-1.087±3.930	6.729±4.284	0.110	0.163

Data were presented as estimated mean ± standard error. Sensitivity analyses were conducted by multivariable general linear model (GLM/ANCOVA) with the adjusted covariates including age (yrs), gender (female/male), baseline BMI (kg/m^2), medication usages for hypertension (yes/no), medical history of thyroid dysfunction (yes/no), change of total physical activity (Met-h/week), change of energy-adjusted dietary protein intakes (g/1000kcal/d), smoking (yes/no) and alcohol drinking (yes/no). and baseline value of the tested variable for change (not for change%). *Analysis was made by k-mean Kruskal-Wallis test and trend analysis by Jonckheere-Terpstra test due to notable variable heterogeneity (p<0.001). Change=final value-baseline value; Change%=(final value-baseline value)*100%/baseline value.

Table S4 .Subgroup analyses by **gender** (female and male) for the effects of anthocyanins on the pre-post change% of arterial stiffness, four-limb blood pressure and composite cardiovascular markers by ANCOVA

	Anthocyanins supplementation						
	placebo	160mg/d	320mg/d	640mg/d	$P_{overall}$	P_{trend}	$P_{interaction}$
**Female/male, n/n**	37/9	27/19	28/18	33/13			
**Max PWV, cm/s**							
Female	-1.932±2.008	3.340±2.307	-4.445±2.227	-0.755±2.110	0.110	0.730	0.060
Male	-3.392±5.288	0.600±3.788	6.515±3.738	-2.130±4.624	0.356	0.470	
**Min ABI**							
Female	-0.548±1.168	0.631±1.340	-1.359±1.293	-0.087±1.212	0.747	0.664	0.206
Male	1.669±2.350	-1.375±1.637	3.185±1.661	-0.581±2.057	0.236	0.987	
**Left brachial SBP**							
Female	-2.167±1.453	-2.061±1.667	-3.951±1.608	-3.940±1.507	0.712	0.423	0.616
Male	-4.103±2.607	-1.940±1.816	0.027±1.843	-3.327±2.282	0.538	0.754	
**Left brachial DBP**							
Female	-1.558±1.836	-1.080±2.107	-3.482±2.032	-1.214±1.905	0.819	0.920	0.452
Male	-3.781±3.504	0.341±2.440	0.955±2.476	-4.600±3.066	0.424	0.972	
**IAD of SBP, mmHg**							
Female	100.321±37.154	2.345±44.024	20.678±42.233	124.854±39.935	0.110	0.566	0.133
Male	-5.444±67.179	123.229±47.289	10.892±48.882	11.656±66.262	0.281	0.874	
**IAND of SBP, mmHg**							
Female	83.547±48.720	55.844±60.483	104.204±57.699	32.544±51.066	0.785	0.484	0.631
Male	-31.859±102.088	86.990±72.669	35.288±74.076	74.574±89.058	0.798	0.677	
**RC, mmol/L**							
Female	14.654±14.233	9.073±17.174	-19.276±15.846	47.074±14.660	0.026	0.102	0.149

Male	6.155±50.549	83.177±35.330	5.085±36.707	44.488±44.169	0.428	0.814	
**Non-HDL, mmol/L**							
Female	1.437±2.147	3.808±2.515	-0.325±2.379	-0.706±2.231	0.538	0.284	0.072
Male	-6.980±5.690	12.385±3.962	4.855±4.021	1.753±4.979	0.053	0.356	
**TG/HDL-c**							
Female	14.094±6.798	-6.520±7.798	-4.366±7.523	-3.556±7.050	0.158	0.247	0.365
Male	-3.354±15.179	21.477±10.569	-7.805±10.727	8.748±13.282	0.254	0.860	
**TyG-index**							
Female	0.564±0.638	-0.380±0.732	-0.579±0.706	-0.558±0.662	0.576	0.399	0.721
Male	-0.364±1.239	1.126±0.863	-1.376±0.876	0.164±1.084	0.250	0.610	
**TyG-BMI**							
Female	0.637±0.819	0.268±0.939	-0.325±0.906	-0.727±0.849	0.681	0.289	0.818
Male	-1.677±1.597	0.119±1.112	-1.821±1.129	0.450±1.398	0.474	0.938	
**VAI**							
Female	15.425±6.893	-7.313±7.907	-6.292±7.628	-4.887±7.148	0.089	0.151	0.380
Male	-3.165±15.066	20.053±10.490	-8.536±10.647	5.347±13.183	0.281	0.747	
**FRS**							
Female	-1.473±3.796	10.256±4.446	-1.003±4.205	-1.155±3.943	0.163	0.698	0.110
Male	-3.290±7.629	2.338±5.301	1.871±5.393	14.187±6.976	0.382	0.083	

Data analyses were conducted by multivariable general linear models (GLM, ANCOVA) and presented as estimated mean±standard error. The adjusted covariates including age (yrs), BMI (kg/m^2), medication usages for hyperglycemia (yes/no), hypertension (yes/no), and thyroid conditions (yes/no). Post-hos comparison was made by LSD approach if overall comparison was significant. Abbreviations: PWV, pulse wave velocity; ABI, ankle brachial index; IAD, systolic inter-arm difference; IAND, inter-ankle difference; SBP, systolic blood pressure; DBP, diastolic blood pressure; RC, remnant cholesterol; TyG-index, triglyceride-glucose index; BMI, body mass index; VAI, visceral adiposity index; FRS, Framingham risk score.

Change=final value-baseline value; change%=(final value-baseline value)*100%/baseline value. The maximum PWV was determined by the higher either left or right PWV. The minimal ABI was estimated by dividing the lower one of ankle SBP with the higher one of brachial SBP. RC = TC-HDL-LDL; Non-HDL= LDL+1/5*TG; TyG-index=In(TG(mg/dl)*FG(mg/dl)/2); TyG-BMI=TyG-index*BMI; Visceral adiposity index (VAI)= WC/(39.68+1.88*BMI)*(TG/1.03)*(1.31/HDL-c) (for males), and WC/(36.58+1.89*BMI)*(TG/0.81)*(1.52/HDL-c) for female.

Table S5. Subgroup analyses by **age (<65 and ≥65 yrs)** for the effects of anthocyanins on the pre-post change% of arterial stiffness, four-limb blood pressure and composite cardiovascular markers by ANCOVA

	Anthocyanins supplementation						
	placebo	160mg/d	320mg/d	640mg/d	$P_{overall}$	P_{trend}	$P_{interaction}$
**Age<65/≥65 yrs, n/n**	22/24	18/28	19/27	26/20			
**Max PWV, cm/s**							
age<65	-3.736±2.266	2.112±2.583	-1.326±2.454	0.187±2.090	0.383	0.375	0.803
age≥65	-1.733±3.413	1.988±3.054	-0.080±3.077	-0.284±3.834	0.886	0.842	
**Min ABI**							
age<65	0.944±1.521	0.634±1.689	-0.327±1.645	-1.070±1.401	0.763	0.444	0.708
age≥65	-0.293±1.465	-0.920±1.305	0.487±1.318	0.804±1.604	0.810	0.561	
**Left brachial SBP**							
age<65	-1.901±1.702	-2.769±1.891	-0.476±1.841	-1.988±1.568	0.854	0.879	0.689
age≥65	-3.130±1.817	-1.584±1.619	-3.828±1.635	-5.889±1.989	0.398	0.332	
**Left brachial DBP**							
age<65	-0.509±2.270	-0.077±2.521	0.318±2.455	0.609±2.090	0.986	0.896	0.915
age≥65	-2.797±2.295	-1.308±2.045	-2.901±2.066	-6.089±2.513	0.531	0.768	
**IAD of SBP, mmHg**							
age<65	139.366±56.465	97.936±66.861	40.843±61.224	73.930±54.372	0.674	0.244	0.094
age≥65	0 (-60.0, 100.0)	0(-50.0, 57.8)	-12.5(-61.2, 25.0)	0(-50.0, 325.0)	0.455	0.706*	
**IAND of SBP, mmHg**							
age<65	-1.282±52.877	-14.564±61.235	154.116±60.318	62.032±47.339	0.170	0.168	0.081
age≥65	147.722±66.808	113.452±63.129	33.973±62.714	-19.305±74.654	0.300	0.133	

**RC, mmol/L**							
age<65	8.431±16.068	41.807±18.419	-14.808±17.397	38.137±14.785	0.081	0.711	0.981
age≥65	30.341±29.668	31.339±27.053	-9.228±27.306	59.524±32.110	0.451	0.528	
**Non-HDL, mmol/L**							
age<65	2.129±2.818	7.044±3.130	-1.261±3.048	0.620±2.596	0.269	0.414	0.342
age≥65	-2.092±3.308	7.838±2.948	3.394±2.977	-0.854±3.621	0.122	0.964	
**TG/HDL-c**							
age<65	5.027±8.191	13.868±9.099	-7.048±8.860	-4.090±7.544	0.322	0.131	0.564
age≥65	13.871±9.705	-4.104±8.646	-2.075±8.734	8.751±10.633	0.483	0.729	
**TyG-index**							
age<65	0.254±0.769	0.371±0.855	-1.306±0.832	-1.054±0.709	0.315	0.064	0.580
age≥65	0.155±0.867	0.103±0.772	-0.348±0.780	0.710±0.949	0.871	0.817	
**TyG-BMI**							
age<65	-0.116±1.090	0.026±1.211	-2.087±1.180	-0.950±1.004	0.557	0.211	0.675
age≥65	0.037±1.036	0.710±0.923	0.281±0.932	-0.200±1.134	0.930	0.914	
**VAI**							
age<65	6.368±7.975	13.421±8.859	-8.805±8.627	-6.197±7.346	0.202	0.061	0.521
age≥65	15.038±9.821	-5.707±8.750	-3.042±8.838	6.595±10.750	0.408	0.618	
**FRS**							
age<65	-2.891±6.744	11.745±7.515	-4.439±7.298	11.760±6.352	0.207	0.344	0.114
age≥65	-1.863±2.443	2.975±2.223	1.455±2.198	-3.381±2.682	0.241	0.236	

Data analyses were conducted by multivariable general linear models (GLM, ANCOVA) and presented as estimated mean±standard error. The adjusted covariates included gender (female vs. male), BMI (kg/m^2), change of TPA (Met-h/week), medication usages for hyperglycemia (yes vs. no), hypertension (yes vs. no), and thyroid conditions (yes vs. no), change of energy-adjusted dietary protein intakes (g/d). Post-hos comparison

was made by LSD approach if overall significance was noted ($P_{overall}$< 0.05). Change%=(final value-baseline value)*100%/baseline value. *P<0.05 compared with placebo group by LSD test. Abbreviations: PWV, pulse wave velocity; ABI, ankle brachial index; IAD, systolic inter-arm difference; IAND, inter-ankle difference; SBP, systolic blood pressure; DBP, diastolic blood pressure; RC, remnant cholesterol; TyG-index, triglyceride-glucose index; BMI, body mass index; VAI, visceral adiposity index; FRS, Framingham risk score. The maximum PWV was determined by the higher either left or right PWV. The minimal ABI was estimated by dividing the lower one of ankle SBP with the higher one of brachial SBP. RC = TC-HDL-LDL; Non-HDL= LDL+1/5*TG; TyG-index=In(TG(mg/dl)*FG(mg/dl)/2); TyG-BMI=TyG-index*BMI; Visceral adiposity index (VAI)= WC/(39.68+1.88*BMI)*(TG/1.03)*(1.31/HDL-c) (for males), and WC/(36.58+1.89*BMI)*(TG/0.81)*(1.52/HDL-c) for female.

Table S6. Subgroup analyses by body mass index (BMI <24 vs. ≥24 kg/m^2) for the effects of anthocyanins on the pre-post change% of arterial stiffness, four-limb blood pressures and composite cardiovascular markers by ANCOVA

	Anthocyanins supplementation						
	placebo	160mg/d	320mg/d	640mg/d	$P_{overall}$	P_{trend}	$P_{interaction}$
**BMI<24/≥24 kg/m^2 (n/n)**	16/30	22/24	23/23	26/20			
**Max PWV, cm/s**							
BMI<24	-0.942±2.407	1.495±2.081	-1.873±2.007	-0.836±1.950	0.690	0.918	0.777
BMI≥24	-2.759±3.173	2.071±3.545	1.129±3.544	-0.237±3.892	0.775	0.770	
**Min ABI**							
BMI<24	1.961±1.691	-1.732±1.464	-0.991±1.414	-0.219±1.355	0.434	0.608	0.553
BMI≥24	-0.314±1.336	0.387±1.458	0.753±1.490	0.629±1.635	0.953	0.840	
**Left brachial SBP**							
BMI<24	-0.090±2.102	-1.077±1.820	-3.073±1.758	-3.644±1.684	0.487	0.099	0.342
BMI≥24	-3.400±1.594	-3.126±1.740	-2.308±1.778	-3.617±1.951	0.962	0.613	
**Left brachial DBP**							
BMI<24	0.285±2.636	0.481±2.283	-3.926±2.204	-0.975±2.112	0.593	0.396	0.352
BMI≥24	-2.558±2.066	-1.235±2.256	-1.098±2.305	-2.061±2.529	0.964	0.628	
**IAD of SBP, mmHg**							
BMI<24	66.010±48.347	-2.825±45.266	17.864±43.114	102.319±42.727	0.407	0.595	0.772
BMI≥24	79.918±45.220	91.915±48.704	7.235±49.722	119.011±55.923	0.470	0.930	
**IAND of SBP, mmHg**							
BMI<24	14.528±71.036	150.181±65.377	73.308±63.572	-2.563±56.726	0.377	0.874	0.131
BMI≥24	106.686±57.254	-3.873±65.308	113.876±65.441	33.104±70.694	0.493	0.862	
**RC, mmol/L**							

BMI<24	12.553±37.230	48.801±34.752	-13.485±31.410	78.228±29.115	0.174	0.571	0.752
BMI≥24	7.806±14.767	35.818±16.119	-13.969±16.475	22.001±18.072	0.170	0.949	
**Non-HDL, mmol/L**							
BMI<24	-0.874±3.212	4.963±2.781	-0.860±2.685	2.343±2.574	0.390	0.751	0.651
BMI≥24	-0.162±2.911	9.320±3.177	2.531±3.248	-0.185±3.562	0.134	0.404	
**TG/HDL-c**							
BMI<24	18.813±11.101	3.534±9.612	-0.938±9.281	-2.281±8.895	0.470	0.111	0.578
BMI≥24	1.901±7.782	7.134±8.495	-4.416±8.683	1.619±9.524	0.817	0.682	
**TyG-index**							
BMI<24	0.383±0.973	0.446±0.842	-0.414±0.813	-0.174±0.780	0.856	0.465	0.987
BMI≥24	0.113±0.739	0.066±0.807	-1.046±0.825	-0.568±0.905	0.695	0.313	
**TyG-BMI**							
BMI<24	0.159±1.183	0.489±1.024	0.517±0.989	-0.327±0.948	0.931	0.699	0.637
BMI≥24	0.031±0.958	0.158±1.046	-2.336±1.069	-0.487±1.173	0.309	0.269	
**VAI**							
BMI<24	21.535±11.213	3.495±9.710	-3.296±9.375	-4.130±8.985	0.280	0.058	0.482
BMI≥24	2.068±7.709	5.319±8.415	-4.663±8.601	-0.826±9.435	0.855	0.562	
**FRS**							
BMI<24	-1.115±4.543	1.153±4.045	-2.407±3.804	4.412±3.648	0.605	0.346	0.479
BMI≥24	1.311±4.896	10.584±5.356	0.811±5.471	-0.684±6.128	0.457	0.857	

Data were presented as estimated mean±standard error. Subgroup analyses were conducted by multivariable general linear model (GLM) with the adjusted covariates including age (yrs), gender (female/male), change of total physical activity (Met-h/d), medication usages for hyperglycemia (yes/no), hypertension (yes /no), and thyroid conditions (yes/no), change of energy-adjusted dietary protein intakes (g/1000kcal/d). Post-hos comparison was made by LSD approach if overall significance was noted ($P_{overall}$< 0.05). Change%=(final value-baseline value)*100%/baseline

value. Abbreviations: PWV, pulse wave velocity; ABI, ankle brachial index; IAD, systolic inter-arm difference; IAND, inter-ankle difference; SBP, systolic blood pressure; DBP, diastolic blood pressure; RC, remnant cholesterol; TyG-index, triglyceride-glucose index; BMI, body mass index; VAI, visceral adiposity index; FRS, Framingham risk score. The maximum PWV was determined by the higher either left or right PWV. The minimal ABI was estimated by dividing the lower one of ankle SBP with the higher one of brachial SBP. RC=TC-HDL-LDL; Non-HDL= LDL+1/5*TG; TyG-index=In(TG(mg/dl)*FG(mg/dl)/2); TyG-BMI=TyG-index*BMI; Visceral adiposity index (VAI)= WC/(39.68+1.88*BMI)*(TG/1.03)*(1.31/HDL-c) (for males), and WC/(36.58+1.89*BMI)*(TG/0.81)*(1.52/HDL-c) for female.

Table S7. Subgroup analyses by **severity of hyperglycemia (pre-diabetes vs. diabetes)** for the effects of anthocyanins on the pre-post change% of arterial stiffness, four-limb blood pressures and composite cardiovascular markers by ANCOVA

	Anthocyanins supplementation						
	placebo	160mg/d	320mg/d	640mg/d	$P_{overall}$	P_{trend}	$P_{interaction}$
**Pre-DM/DM, n/n**	20/26	23/23	23/23	23/23			
**Max PWV, cm/s**							
pre-DM	-3.301±2.544	-0.696±2.322	-0.435±2.310	-1.926±2.338	0.841	0.643	0.971
DM	-2.963±3.064	5.266±3.292	-0.726±3.215	2.459±3.282	0.322	0.856	
**Min ABI**							
pre-DM	0.566±1.639	-0.323±1.496	1.463±1.488	-0.989±1.506	0.674	0.589	0.623
DM	-0.100±1.407	-0.209±1.476	-0.885±1.465	0.326±1.479	0.952	0.719	
**Left brachial SBP**							
pre-DM	-3.523±1.640	-2.168±1.497	-2.510±1.489	-6.076±1.508	0.266	0.395	0.448
DM	-1.692±1.920	-1.730±2.014	-2.408±1.998	-1.568±2.018	0.991	0.894	
**Left brachial DBP**							
pre-DM	-2.570±2.158	-1.831±1.970	-1.646±1.959	-4.060±1.983	0.824	0.960	0.675
DM	-1.328±2.430	1.321±2.549	-2.096±2.529	-0.758±2.554	0.809	0.869	
**IAD of SBP, mmHg**							
pre-DM	121.984±49.963	77.074±48.370	19.778±45.659	77.645±48.839	0.526	0.722	0.896
DM	44.457±43.575	36.353±45.021	-1.285±48.050	124.682±48.679	0.338	0.463	
**IAND of SBP, mmHg**							
pre-DM	35.542±70.684	57.616±67.494	103.254±68.261	66.626±64.728	0.921	0.811	0.723
DM	82.300±57.383	71.868±63.913	45.159±61.434	32.793±60.927	0.930	0.318	
**RC, mmol/L**							

pre-DM	9.183±16.187	6.987±15.346	-10.458±14.768	48.764±14.588	0.040	0.142	0.209
DM	26.959±28.494	69.800±30.650	-17.702±30.361	44.305±30.074	0.243	0.972	
**Non-HDL, mmol/L**							
pre-DM	-2.009±2.856	6.023±2.607	7.922±2.594	1.941±2.625	0.063	0.767	0.317
DM	-1.053±2.917	7.492±3.060	-2.295±3.036	-0.337±3.065	0.110	0.352	
**TG/HDL-c**							
pre-DM	5.856±8.408	-4.142±7.675	-3.116±7.636	2.410±7.728	0.801	0.959	0.450
DM	13.651±9.628	10.809±10.100	-8.585±10.020	1.967±10.118	0.382	0.138	
**TyG-index**							
pre-DM	0.180±0.792	-0.665±0.723	-0.469±0.719	-0.502±0.728	0.877	0.739	0.445
DM	0.466±0.814	0.903±0.854	-1.126±0.847	-0.066±0.856	0.363	0.243	
**TyG-BMI**							
pre-DM	0.142±0.968	-0.431±0.884	-0.471±0.880	-0.983±0.890	0.869	0.316	0.642
DM	0.046±1.071	0.745±1.124	-0.698±1.115	-0.166±1.126	0.836	0.590	
**VAI**							
pre-DM	6.000±8.359	-4.599±7.631	-5.770±7.591	1.308±7.683	0.717	0.814	0.478
DM	15.696±9.739	9.206±10.215	-8.871±10.135	-1.009±10.234	0.315	0.082	
**FRS**							
pre-DM	-4.464±4.389	-1.172±4.099	1.850±3.997	2.937±4.130	0.621	0.126	0.340
DM	-0.790±5.063	13.855±5.311	0.537±5.269	2.703±5.321	0.209	0.880	

Data were presented as estimated mean±standard error. Subgroup analyses were conducted by multivariable general linear model (GLM) with the adjusted covariates including age (yrs), gender, BMI (kg/m^2), medication usages for hyperglycemia (yes/no), hypertension (yes/no), and thyroid conditions (yes/no), change of energy-adjusted dietary protein intakes (g/1000kcal/d). Post-hos comparison was made by LSD approach if overall significance was noted ($P_{overall}< 0.05$). Change%=(final value-baseline value)*100%/baseline value. Abbreviations: DM, diabetes; pre-

DM, prediabetes; PWV, pulse wave velocity; ABI, ankle brachial index; IAD, systolic inter-arm difference; IAND, inter-ankle difference; SBP, systolic blood pressure; DBP, diastolic blood pressure; RC, remnant cholesterol; TyG-index, triglyceride-glucose index; BMI, body mass index; VAI, visceral adiposity index; FRS, Framingham risk score. The maximum PWV was determined by the higher either left or right PWV. The minimal ABI was estimated by dividing the lower one of ankle SBP with the higher one of brachial SBP. RC = TC-HDL-LDL; Non-HDL= LDL+1/5*TG; TyG-index=In(TG(mg/dl)*FG(mg/dl)/2); TyG-BMI=TyG-index*BMI; Visceral adiposity index (VAI)= WC/(39.68+1.88*BMI)*(TG/1.03)*(1.31/HDL-c) (for males), and WC/(36.58+1.89*BMI)*(TG/0.81)*(1.52/HDL-c) for female.

Table S8. Subgroup analyses by **baseline hypertension** for the effects of anthocyanins on the pre-post change% of arterial stiffness, four-limb blood pressure and composite cardiovascular markers by ANCOVA

	Anthocyanins supplementation						
	placebo	160mg/d	320mg/d	640mg/d	$P_{overall}$	P_{trend}	$P_{interaction}$
Norm-/hypertension(n/n)	27/19	28/18	26/20	31/15			
**Max PWV, cm/s**							
Norm-tension	-0.320±2.449	-0.833±2.406	0.746±2.446	-1.591±2.316	0.920	0.992	0.410
hypertension	-6.565±3.605	8.261±3.710	-1.814±3.238	0.778±3.913	0.061	0.560	
**Min ABI**							
Norm-tension	-0.171±1.431	-0.094±1.383	0.065±1.434	-0.439±1.347	0.995	0.958	0.939
hypertension	0.490±1.707	-0.396±1.757	0.984±1.533	-0.544±1.853	0.903	0.824	
**Left brachial SBP**							
Norm-tension	-0.045±1.410	-1.655±1.363	-2.256±1.413	-4.000±1.328	0.250	0.143	0.539
hypertension	-6.255±2.258	-1.447±2.324	-3.574±2.028	-3.097±2.451	0.569	0.551	
**Left brachial DBP**							
Norm-tension	-0.017±1.950	0.861±1.885	-1.613±1.955	-1.522±1.837	0.748	0.593	0.825
hypertension	-5.641±2.823	-1.812±2.906	-2.948±2.536	-2.023±3.065	0.792	0.623	
**IAD of SBP, mmHg**							
Norm-tension	62.219±42.067	40.193±42.513	15.477±42.791	154.663±42.373	0.127	0.469	0.623
hypertension	58.089±52.584	64.497±52.937	17.453±49.138	45.848±60.496	0.909	0.522	
**IAND of SBP, mmHg**							
Norm-tension	56.280±50.197	9.640±51.602	51.093±50.317	33.536±47.352	0.917	0.725	0.786
hypertension	61.678±87.936	206.489±97.930	77.594±90.529	52.077±96.177	0.684	0.734	
**RC, mmol/L**							

Norm-tension	6.178±24.004	58.245±23.803	-5.395±23.676	38.836±22.319	0.211	0.927	0.411
hypertension	14.446±22.404	23.367±24.429	-20.700±21.318	69.405±24.409	0.063	0.162	
**Non-HDL, mmol/L**							
Norm-tension	-0.837±2.694	7.807±2.604	0.528±2.700	-2.797±2.537	0.029	0.196	0.441
hypertension	-1.308±3.718	8.597±3.827	4.395±3.340	4.587±4.036	0.383	0.333	
**TG/HDL-c**							
Norm-tension	-1.612±7.133	2.746±6.894	-6.218±7.148	-2.728±6.717	0.838	0.655	0.684
hypertension	27.286±12.951	3.886±13.328	-2.744±11.633	9.088±14.059	0.404	0.282	
**TyG-index**							
Norm-tension	-0.268±0.701	-0.027±0.677	-1.059±0.702	-1.019±0.660	0.619	0.333	0.979
hypertension	0.993±1.061	0.713±1.092	-0.238±0.953	0.774±1.152	0.819	0.677	
**TyG-BMI**							
Norm-tension	-0.784±0.912	0.524±0.882	-1.167±0.914	-1.486±0.859	0.393	0.313	0.698
hypertension	0.909±1.322	0.139±1.361	-0.033±1.188	1.450±1.436	0.862	0.867	
**VAI**							
Norm-tension	-1.141±7.044	2.009±6.807	-8.160±7.059	-4.095±6.632	0.756	0.494	0.637
hypertension	29.339±13.174	2.206±13.559	-3.622±11.834	6.211±14.302	0.324	0.192	
**FRS**							
Norm-tension	-1.938±4.555	8.675±4.402	-1.127±4.564	0.282±4.289	0.312	0.950	0.722
hypertension	0.137±5.311	2.610±5.741	1.800±4.779	7.979±6.037	0.795	0.227	

Data were presented as estimated mean±standard error. Baseline hypertension was defined as left brachial BP with either SBP>140 or DBP>90 mmHg or under medication treatment for lowering blood pressure. Subgroup analyses were conducted by multivariable general linear model (GLM) with the adjusted covariates including age (yrs), gender, BMI (kg/m^2), medication usages for hyperglycemia (yes/no), hypertension (yes/no), and thyroid conditions (yes/no), medical history of cardiovascular diseases (yes/no). Post-hos comparison was made by LSD approach

if overall significance was noted ($P_{overall} < 0.05$). Change%=(final value-baseline value)*100%/baseline value. *P<0.05 compared with placebo group by LSD test. Abbreviations: PWV, pulse wave velocity; ABI, ankle brachial index; IAD, systolic inter-arm difference; IAND, inter-ankle difference; SBP, systolic blood pressure; DBP, diastolic blood pressure; RC, remnant cholesterol; TyG-index, triglyceride-glucose index; BMI, body mass index; VAI, visceral adiposity index; FRS, Framingham risk score. The maximum PWV was determined by the higher either left or right PWV. The minimal ABI was estimated by dividing the lower one of ankle SBP with the higher one of brachial SBP. RC = TC-HDL-LDL; Non-HDL= LDL+1/5*TG; TyG-index=In(TG(mg/dl)*FG(mg/dl)/2); TyG-BMI=TyG-index*BMI; Visceral adiposity index (VAI)= WC/(39.68+1.88*BMI)*(TG/1.03)*(1.31/HDL-c) (for males), and WC/(36.58+1.89*BMI)*(TG/0.81)*(1.52/HDL-c) for female.
Baseline hypertension was defined as left brachial BP with either SBP>140 or DBP>90 mmHg or under medication treatment for lowering blood pressure.

Table S9. Subgroup analyses by **baseline hyperlipidemia** for the effects of anthocyanins on the pre-post change% of arterial stiffness, four-limb blood pressures and composite cardiovascular markers by ANCOVA

	Anthocyanins supplementation						
	placebo	160mg/d	320mg/d	640mg/d	$P_{overall}$	P_{trend}	$P_{interaction}$
Norm-/hyper-lipidemia(n/n)	20/26	30/16	20/26	23/23			
**Max PWV, cm/s**							
norm-lipidemia	-0.612±2.510	1.384±1.994	-1.198±2.408	1.126±2.322	0.828	0.310	0.739
hyperlipidemia	-2.854±3.109	2.362±3.941	0.137±3.066	-2.433±3.365	0.726	0.763	
**Min ABI**							
norm-lipidemia	-0.771±1.658	-1.011±1.298	0.845±1.588	1.833±1.506	0.476	0.332	0.349
hyperlipidemia	0.098±1.390	1.788±1.761	-0.168±1.370	-1.980±1.504	0.461	0.229	
**Left brachial SBP**							
norm-lipidemia	1.298±2.157	-1.622±1.688	-1.915±2.066	-3.809±1.958	0.409	0.219	0.476
hyperlipidemia	-4.649±1.499	-3.273±1.899	-2.781±1.478	-4.298±1.622	0.822	0.620	
**Left brachial DBP**							
norm-lipidemia	3.152±2.521	1.227±1.973	1.792±2.414	-3.516±2.288	0.231	0.265	0.140
hyperlipidemia	-4.614±2.052	-4.723±2.601	-4.758±2.024	-1.310±2.221	0.643	0.202	
**IAD of SBP, mmHg**							
norm-lipidemia	66.880±48.732	88.920±39.223	-29.970±46.904	143.893±48.567	0.081	0.934	0.505
hyperlipidemia	83.411±44.501	-11.109±57.960	54.153±46.033	61.097±49.045	0.645	0.628	
**IAND of SBP, mmHg**							
norm-lipidemia	124.953±84.114	12.122±69.347	177.790±82.663	79.833±76.383	0.480	0.871	0.067
hyperlipidemia	47.359±44.310	131.101±58.664	-18.515±46.328	11.525±48.004	0.228	0.127	
**RC, mmol/L**							

norm-lipidemia	45.680±36.360	63.546±28.950	-10.959±35.871	93.047±32.437	0.202	0.452	0.110
hyperlipidemia	-0.330±7.483	-8.164±9.782	-11.513±7.374	-3.514±8.081	0.750	0.960	
**Non-HDL, mmol/L**							
norm-lipidemia	3.297±3.512	8.189±2.749	4.463±3.364	2.683±3.188	0.549	0.795	0.971
hyperlipidemia	-3.185±2.751	5.640±3.486	-0.848±2.712	-1.665±2.976	0.258	0.680	
**TG/HDL-c**							
norm-lipidemia	16.525±9.553	9.823±7.476	5.238±9.149	10.880±8.672	0.865	0.260	0.923
hyperlipidemia	3.401±8.391	-5.514±10.634	-16.196±8.275	-4.448±9.080	0.439	0.493	
**TyG-index**							
norm-lipidemia	1.163±0.884	0.851±0.692	0.188±0.847	0.417±0.802	0.851	0.249	0.887
hyperlipidemia	-0.586±0.716	-0.715±0.908	-1.710±0.706	-0.851±0.775	0.688	0.621	
**TyG-BMI**							
norm-lipidemia	1.196±1.259	0.613±0.986	-0.125±1.206	0.561±1.143	0.901	0.479	0.709
hyperlipidemia	-1.112±0.790	0.207±1.001	-1.256±0.779	-1.588±0.854	0.572	0.339	
**VAI**							
norm-lipidemia	18.360±9.344	8.568±7.313	3.335±8.950	8.047±8.483	0.709	0.147	0.899
hyperlipidemia	4.662±8.617	-6.396±10.921	-17.597±8.498	-5.791±9.325	0.353	0.397	
**FRS**							
norm-lipidemia	-4.774±5.843	9.454±4.573	0.513±5.597	10.084±5.305	0.187	0.147	0.368
hyperlipidemia	0.684±3.604	1.659±4.718	-3.034±3.558	-1.337±3.990	0.832	0.569	

Data were presented as estimated mean±standard error. Subgroup analyses were conducted by multivariable general linear model (GLM) with the adjusted covariates including age (yrs), gender (female/male), BMI (kg/m^2), medication usages for hyperglycemia (yes/no), hypertension (yes/no), and thyroid conditions (yes/no), change of energy-adjusted dietary protein intakes (g/1000kcal/d). Post-hos comparison was made by

LSD approach if overall significance was noted ($P_{overall} < 0.05$). Hyperlipidemia was defined as at least one lipid level higher than the following cutoffs for the normal ranges (TG≥2.3, TC≥6.2, LDL-c≥4.1, or HDL<1.0 mmol/L) or under lipid-lowering therapy.
